# Not1 and Not4 inversely determine mRNA solubility that sets the dynamics of co-translational events

**DOI:** 10.1186/s13059-023-02871-7

**Published:** 2023-02-20

**Authors:** George Allen, Benjamin Weiss, Olesya O. Panasenko, Susanne Huch, Zoltan Villanyi, Benjamin Albert, Daniel Dilg, Marina Zagatti, Paul Schaughency, Susan E. Liao, Jeff Corden, Christine Polte, David Shore, Zoya Ignatova, Vicent Pelechano, Martine A. Collart

**Affiliations:** 1 Departement of Microbiology and Molecular Medicine, Institute of Genetics and Genomics Geneva, Faculty of Medicine, University of Geneva, Geneva, Switzerland; 2 Present address: Department of Biomolecular Sciences, The Weizmann Institute of Science, 76100 Rehovot, Israel; 3 SciLifeLab, Department of Microbiology, Tumor and Cell Biology, Karolinska Institutet, Solna, Sweden; 4 Department of Biochemistry and Molecular Biology, Faculty of Science and Informatics, University of Szeged, Szeged, Hungary; 5 Department of Molecular and Cellular Biology, Faculty of Sciences, Institute of Genetics and Genomics Geneva, University of Geneva, Geneva, Switzerland; 6 Present Address: Molecular, Cellular & Developmental Biology (MCD), Center for Integrative Biology (CBI), University of 11, CNRS/UPS, Bâtiment IBCG, 118, Route de Narbonne, 31062 ToulouseToulouse Cedex 9, France; 7 Department of Molecular Biology and Genetics, The Johns Hopkins University School of Medicine, Baltimore, MD USA; 8 Present Address: Axle Informatics, NIAID Collaborative Bioinformatics Resource, North Bethesda, MD USA; 9 Present Address: Department of Computer Science, Courant Institute of Mathematical Sciences, New York University, New York, USA; 10 Departement of Biochemistry and Molecular Biology, University of Hamburg, Hamburg, Germany

**Keywords:** Not1, Not4, mRNA solubility, Condensation, Translation elongation dynamics, Co-translational mRNA decay, Codon optimality, mRNA stability

## Abstract

**Background:**

The Ccr4-Not complex is mostly known as the major eukaryotic deadenylase. However, several studies have uncovered roles of the complex, in particular of the Not subunits, unrelated to deadenylation and relevant for translation. In particular, the existence of Not condensates that regulate translation elongation dynamics has been reported. Typical studies that evaluate translation efficiency rely on soluble extracts obtained after the disruption of cells and ribosome profiling. Yet cellular mRNAs in condensates can be actively translated and may not be present in such extracts.

**Results:**

In this work, by analyzing soluble and insoluble mRNA decay intermediates in yeast, we determine that insoluble mRNAs are enriched for ribosomes dwelling at non-optimal codons compared to soluble mRNAs. mRNA decay is higher for soluble RNAs, but the proportion of co-translational degradation relative to the overall mRNA decay is higher for insoluble mRNAs. We show that depletion of Not1 and Not4 inversely impacts mRNA solubilities and, for soluble mRNAs, ribosome dwelling according to codon optimality. Depletion of Not4 solubilizes mRNAs with lower non-optimal codon content and higher expression that are rendered insoluble by Not1 depletion. By contrast, depletion of Not1 solubilizes mitochondrial mRNAs, which are rendered insoluble upon Not4 depletion.

**Conclusions:**

Our results reveal that mRNA solubility defines the dynamics of co-translation events and is oppositely regulated by Not1 and Not4, a mechanism that we additionally determine may already be set by Not1 promoter association in the nucleus.

**Supplementary Information:**

The online version contains supplementary material available at 10.1186/s13059-023-02871-7.

## Background

Adequate regulation of gene expression is essential for health, fitness, and development of all living organisms. While transcription is the most immediate and focal point of gene regulation, gene expression is also importantly controlled at post-transcriptional levels [1–4]. Repression of translation initiation, the major rate-limiting step of translation, for instance, plays a key role in cellular responses to nutrient levels and stresses [5–7]. Nevertheless, translation output can also be regulated at the elongation step, according to the availability of charged tRNAs, codon bias, the amino acid composition of the nascent chain, co-translational folding, interactions of nascent chains with auxiliary factors, and by mRNA localization or mRNA partitioning into membrane-less granules [8–14]. mRNA-protein condensates were first associated with translational repression (stress granules) and mRNA decay (p-bodies) [15], but recent evidence indicates active translation in stress granules [16], and positive roles of granules for translation have been proposed [12, 17–19]. The development of techniques such as ribosome profiling (Ribo-Seq) [20], visualizing with codon-specific precision the position of ribosomes on mRNAs genome-wide, or the sequencing of 5′P decay intermediates (5′P-Seq) [21], revealing patterns of co-translational decay intermediates, has enabled the analysis of translation elongation dynamics with unprecedented depth and precision.

The conserved Ccr4-Not complex plays a key role in mRNA metabolism [22]. First identified as a transcriptional regulator [23–25], a role later confirmed [26–30], Ccr4-Not is most well-known as the major eukaryotic deadenylase [31–33]. Thereby, it is central in mRNA turnover and translational repression [34, 35]. It is generally active for post-translational mRNA decay, but can also be tethered to mRNAs by RNA binding proteins or the microRNA machinery [36–39]. Ccr4-Not can also inhibit translation independently of deadenylation [40] or activate decapping [41].

Ccr4-Not additionally regulates translation and co-translational processes. Ribosome-associated proteins, notably the nascent polypeptide ribosome-associated complex (NAC) and Rps7A [42, 43], are known targets of Not4 ubiquitination. The ubiquitination-deubiquitination cycles of Rps7A are important for translation [44] and non-ubiquitinated Rps7A enables translation elongation through polyarginine stretches that normally provoke ribosome stalling [17]. Rps7A ubiquitination is also important for translation regulation during ER stress [45]. Not proteins co-sediment with polysomes [42, 46] and Not5 polysome-association is promoted by Rps7A ubiquitination. Furthermore, the co-translational association of proteins is impaired in the absence of Not4 or Not5 [47–49] and co-localization of mRNAs encoding two subunits of the proteasome that assemble co-translationally depends upon Not1 [18]. It was recently shown that Not5 associates with the ribosomal E site in post-translocation state providing thereby a means for the Ccr4-Not complex to monitor the translating ribosome according to codon optimality [50]. It was proposed that this regulates the turnover of mRNAs, consistent with Not5-dependent longer half-lives of mRNAs with a high content of non-optimal codons [50]. We recently proposed an alternative role for Not4 and Not5, consistent with Not5 monitoring the translating ribosome according to codon optimality [17]. In our model, we proposed that Not5 can tether ribosome-nascent chain complexes (RNCs) to condensates that exclude the translation initiation and elongation factor eIF5A [51, 52]. A central role of Not4 and Not5 in translation elongation dynamics is corroborated by the fact that, when deleted in cells, newly synthesized proteins massively aggregate [42, 53] accompanied with a high level of abortive translation products [17].

Condensate mRNAs, like other insoluble mRNAs such as membrane-associated mRNAs, are not captured by typical ribosome and polysome profiling approaches. In this work, we used the sequencing of mRNA decay intermediates to address whether soluble mRNAs and insoluble mRNAs have different translation elongation dynamics and if different mRNA classes partition differently into soluble and insoluble mRNA pools. We further investigated if elongation dynamics of the different mRNA pools was altered immediately upon depletion of Not1, Not4, and Not5. Taken together, our data indicate that ribosomes dwell at non-optimal codons in the non-soluble RNA fraction. In turn, soluble mRNAs show more mRNA 5′ to 3′ degradation, but proportionally less co-translational decay. Additionally, we determine that the depletion of Not1 and Not4 regulate mRNA partitioning between soluble and insoluble fractions in an opposing manner, which in turn correlates with the opposite impacts on ribosome dwelling at optimal and non-optimal codons in the soluble RNA pool. Our results are compatible with a model whereby mRNA solubility sets the dynamics of co-translational events and is regulated by the Not proteins.

## Results

### Paused ribosomes at non-optimal codons are enriched within non-soluble mRNA fractions

In a recent study, we showed that the dynamics of translation elongation were greatly affected in the absence of Not4 and Not5 in *Saccharomyces cerevisiae* and we proposed that this was due to defective tethering of translating ribosomes to ribonucleoprotein (RNP) condensates, in particular ribosomes paused at non-optimal codons [17]. This model predicts that ribosomes paused at non-optimal codons should be enriched in the insoluble mRNA condensates. To test this idea, we needed a means to detect insoluble mRNAs. To achieve this, we compared the cell’s total mRNA pool that can be prepared from cell pellets (called hereafter “total RNA”) and includes insoluble mRNAs, and the cell’s soluble mRNA pool obtained from cell lysates (called hereafter “soluble RNA pool”). We investigated the distribution of mRNAs between the soluble and total RNA pools (referred to from here onwards as “solubility”) and the 5′P mRNA decay intermediates in total versus soluble RNA pools. We grew wild-type cells in rich medium to exponential phase and split them in two, extracting the total RNA from one aliquot and the soluble RNA pool from the other, in biological triplicates. For normalization, we spiked in each sample a constant amount of RNA from *S. pombe*. For each sample, one aliquot was subjected to RNA-Seq to determine the transcriptome and the other to 5′P-Seq to determine mRNA decay intermediates that can provide information on co-translational decay and ribosome dwelling [21] (Table S1).

**Fig. 1:**
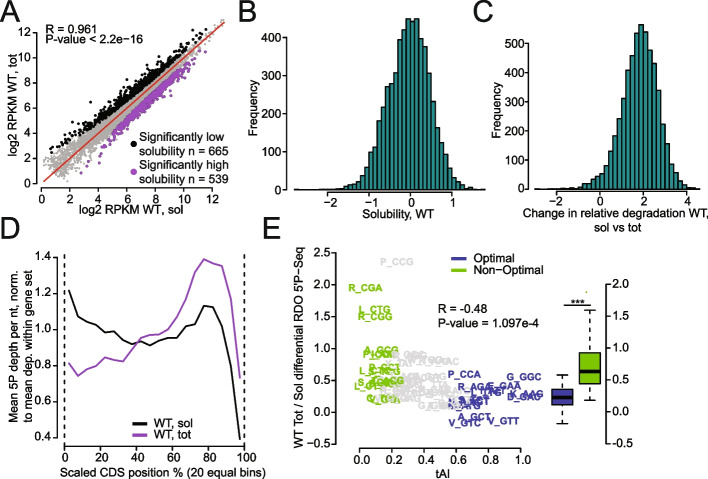
mRNAs that are less soluble are enriched in non-optimal codons. **A** Scatterplot comparing RPKMs of mRNAs in soluble (sol) and total (tot) mRNA pools in WT. mRNAs significantly less soluble after depletion are indicated in black and more soluble in purple (cutoffs from DESeq2 RNA-Seq sol/tot—high solubility: [log2FC > 0, FDR < 0.05] OR [log2FC > 1, *p*-value < 0.05]; low solubility: [log2FC < 0, FDR < 0.05] OR [log2FC <  − 1, *p*-value < 0.05]). **B** Distribution of solubility of mRNAs, defined as log2FC soluble/total from DESeq2 in RNA-Seq. **C** Distribution of relative degradation levels of 5′P mRNA intermediates compared to the RNA abundance (log2FC 5′P-Seq/RNA-Seq from DESeq2 using spike-in) in soluble versus total RNA pools. **D** Metagene profile dividing each CDS into 20 equal bins and finding the mean normalized reads of 5′P mRNA intermediates in each for soluble and total RNA pools in wild type cells. **E** Scatterplot comparing differential 5′P-RDOs in total versus soluble RNA pools with the tRNA adaptation index (tAI). The 15 most optimal codons are indicated in blue, and the 15 most non-optimal codons are shown in green. The optimal and non-optimal codon relative RDOs were compared with a one-sided Wilcoxon rank-sum test giving a *p*-value of 5.627e − 05

We first determined overall mRNA solubilities (log2FC soluble/total). mRNA levels within the soluble RNA pool and total RNA pools correlated overall (Fig. 1A). Nevertheless, the mRNA solubilities spanned a relatively wide range, from − 2.744 to 1.791 (Fig. 1B). It is interesting to note that a GO-term analysis of the mRNAs showing the lowest solubilities revealed “endoplasmic reticulum,” “membrane,” and “cell wall” (Fig. S1A). Membranes are expected to sediment in the first centrifugation step after cell lysis, and mRNAs encoding membrane proteins or proteins that must transit through membranes can be targeted to membranes during translation [12, 54–59]. Hence, such mRNAs can indeed be expected to be depleted from soluble extracts.

To investigate the distribution of 5′P mRNA decay intermediates between soluble and total RNA fractions, we compared the level of mRNA degradation intermediates determined with 5′P-Seq to the overall mRNA level defined with RNA-Seq (Table S1). We used the spike-ins to normalize 5′P-Seq reads to RNA-Seq reads for each RNA pool. This comparison provides a snapshot of the fraction of mRNAs undergoing degradation and we refer to this measure as “relative degradation” from here onwards. The relative degradation was higher in the soluble mRNA pool than in the total RNA pool, as indicated by the shift of distribution between the two pools centered around 2 (Fig. 1C). In addition, metagene profiles of 5′P-Seq depth (i.e., 5′P reads normalized to library size) across coding sequences (CDSs) were significantly different between the soluble and total RNA pools (Fig. 1D). For the soluble RNA pool, we noted high levels of 5′P mRNA ends mapping throughout the CDS, while for the total RNA the 5′P reads were lower at the beginning of the CDS and higher at the end. These metagene profiles were similar for mRNAs with high, medium, or low amount of 5′P reads. The exception were shorter mRNAs that showed no accumulation of 5′P reads at the end of the CDSs (Fig. S1B). For both RNA pools, there was a drop in 5′P reads within the last 150 nucleotides of CDSs, despite our use of a mix of random hexamer and oligo(dT) priming for the library preparation facilitating recovery of regions proximal to the poly(A) site [60]. This is due to the fact that we look at the 5′ region of libraries of a specific insert size. The metagene profile of 5′P decay intermediates for soluble mRNAs was similar to the metagene profile of ribosome footprints observed previously (Fig. S1C) [17].

The difference in metagene profiles of 5′P decay intermediates from soluble and total RNAs could be due to differences in the processivity of 5′ to 3′ decay in the soluble and total mRNA pools, directly or indirectly linked to differences in velocities of ribosomes. Ribosome profiling data provides information on ribosome footprints for the soluble mRNA pool. No similar data can be obtained for insoluble RNAs. However, 5′P-Seq data is informative on ribosome dwelling [21]. Indeed, in the case of co-translational mRNA decay, the progression of the 5′ to 3′ Xrn1 exonuclease can be limited by the dwelling of the last translating ribosome, depending upon relative kinetics of decay and ribosome progression, and the onset of decay compared to progression of the last translating ribosome. Thus, we used 5′P-Seq to compare ribosome dwelling in total and soluble RNA pools. We defined A-site ribosome dwelling occupancies from the 5′P-Seq data (5′P-RDOs), using the 5′P reads 17 nucleotides upstream of each codon. The differential 5′P-RDOs between the total and soluble RNA pools anticorrelated with codon optimality (Fig. 1E), indicating that co-translational decay intermediates accumulating at ribosomes paused on non-optimal codons were enriched in total RNAs compared to soluble RNAs. Since the total RNA pool includes both soluble and insoluble RNAs, these results suggest that ribosome dwelling at non-optimal codons in their A-site is higher in non-soluble RNA fractions. It is relevant to note that the pool of soluble RNAs is likely to differ according to salt and detergent in the lysis buffer. Hence, these findings apply to mRNAs soluble according to standard polysome and ribosome profiling conditions.

mRNA turnover can occur by mechanisms other than co-translational degradation. To get some idea of this for both RNA pools, we generated metagene profiles of mRNA 5′ ends generated by RNA-Seq and compared them to the metagene profiles of 5′P-Seq. The metagene profiles of 5′end reads of the RNA-Seq were more similar between soluble and total RNA pools (Fig. S1D) than the 5′P-Seq metagene profiles (Fig. 1D), particularly there was less difference at the 5′ end of CDSs. This was also apparent by evaluating for soluble and total RNAs reads in the second half of the CDSs (70–90%) to those in the first half (10–30%) for 5′P-Seq and for the 5′end of the RNA-Seq reads (Fig. S1E).

For soluble and total RNAs, the 5′P-RDO changes in the second half of the CDS compared to the first half were inversely correlated with codon optimality (Fig. S1F). These observations could result from the last translating ribosome dwelling longer at non-optimal codons in the second half compared to the first half of CDSs. Alternatively, they could be explained by a delayed decay onset after the last translating ribosome, a model that seems more likely considering that longer mRNAs show more 5′P decay intermediates at the end of CDSs (see above, Fig. S1B).

### Solubility of mRNAs is inversely modified upon depletion of Not1 or Not4

We next investigated the direct role of Not proteins in regulating mRNA solubilities and differences in 5′P-RDOs between total and soluble RNA pools. For this, we created Not4 and Not5 auxin-inducible degron strains, along with the Not1 degron strain described previously [17]. Expression of the degron-regulated proteins was abolished after 15 min of auxin treatment (Fig. S2A). According to multiple replicate experiments, expression of Not4 was not altered after the depletion of Not5, and Not5 expression was not altered after the depletion of Not4, and in both strains, the expression of Not1 was unaffected.

**Fig. 2:**
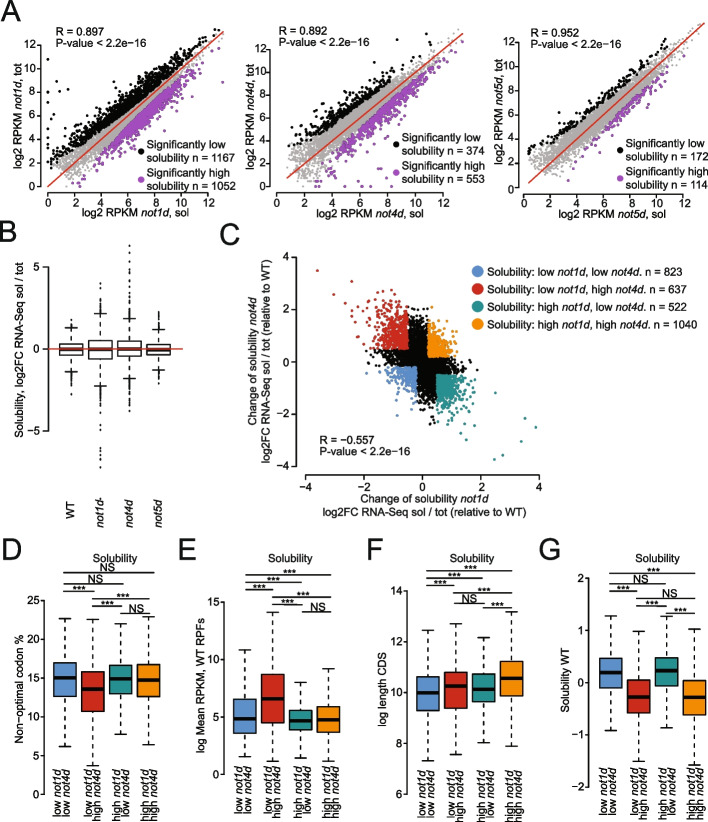
Not1 and Not4 inversely regulate mRNA solubilities. **A** Scatterplot comparing RPKMs of mRNAs in soluble (sol) and total (tot) mRNA pools before and after Not1 (*not1d*), Not4 (*not4d*), and Not5 (*not5d*) depletion (from left to right) in soluble and total mRNA pools. mRNAs significantly less soluble after depletion are indicated in black and more soluble in purple (cutoffs from DESeq2 RNA-Seq sol/tot—high solubility: [log2FC > 0, FDR < 0.05] OR [log2FC > 1, *p*-value < 0.05]; low solubility: [log2FC < 0, FDR < 0.05] OR [log2FC <  − 1, *p*-value < 0.05]). **B** Box plot analysis indicating mRNA solubilities in cells before (WT) or after Not1, Not4, and Not5 depletion. **C** Scatterplot comparing changes in mRNA solubilities before and after Not1 and Not4 depletion. **D–G** Box plot analysis comparing features of mRNAs falling into the four categories defined by changes in mRNA solubilities upon Not1 and Not4 depletion color coded in panel **C** with regard to **D** content in non-optimal codons, **E** abundance (RPF RPKMs), **F** length, and **G** solubility in wild-type cells. Number of mRNAs in box plots, left to right: 823, 637, 522, and 1040. Significance of differences is indicated at the top of the box plots—*p*-values are calculated using a two-sided Welch two-sample t-test

Libraries from soluble and total RNA pools were generated from the degron strains and their isogenic wild-type counterpart following 15 min of auxin treatment (Table S1). As shown above, the expression of mRNAs in total and soluble RNA pools correlated for wild-type cells. However, we noted that the correlation was lower upon Not1 and Not4 depletion, and minimally affected upon Not5 depletion (Fig. 2A). Some mRNAs showed much less solubility upon Not1 depletion and inversely some mRNAs showed much more solubility upon Not4 depletion (Fig. 2B). Interestingly, there was an overall inverse correlation between the changes in mRNA solubility upon Not1 and Not4 depletion (Fig. 2C). The depletion of Not5 exhibited marginal effects on the mRNA solubility and the subtle changes somewhat resembled those observed upon Not4 depletion (Fig. S2B, left panel) but not those observed upon Not1 depletion (Fig. S2B, right panel).

We focused our attention on the mRNA sets that showed opposing changes in solubility following Not1 and Not4 depletion. This concerned 1159 mRNAs which had significantly different solubilities in Not1 and Not4 depletion strains, based on a ratio-of-ratios test in DESeq2 (Table S2). Of these, 637 were less soluble upon Not1 depletion but more soluble upon Not4 depletion and satisfied an additional fold-change cutoff for each strain (solubility not1d/WT <  − 0.5 and not4d/WT > 0.5, red category on Fig. 2C). The remaining 522 mRNAs were more soluble upon Not1 depletion and less soluble upon Not4 depletion (solubility not1d/WT > 0.5 and not4d/WT <  − 0.5, green category on Fig. 2C). We also established a control group of mRNAs which did not have significantly different solubilities between Not1 and Not4 depletion strains, splitting them into two groups of comparable size: increasing (orange category, *n* = 914) and decreasing (blue category, *n* = 702) solubilities in both strains, as we suspected there would be heterogeneity within the control set at these opposing extremes. We checked for specific features of all these mRNA groups, such as their expression level, length and content in non-optimal codons, and solubility in wild type cells (Fig. 2D–G). mRNAs with lowered solubility upon Not1 depletion and instead higher solubility upon Not4 depletion (red category) had a significant lower content in non-optimal codons (Fig. 2D) and were higher expressed (Fig. 2E). mRNAs with higher solubility upon Not1 depletion and instead lower solubility upon Not4 depletion (green category) were enriched for the GO-term “mitochondrial organization” and “mitochondrial translation” (Fig. S2C). mRNAs with reduced solubility upon either Not1 or Not4 depletion (blue category) were distinguishable from those with higher solubility (orange category) by their shorter length (Fig. 2F) and were enriched for the GO-term “endocytosis”, while the mRNAs with higher solubility upon either Not1 or Not4 depletion (orange category) were enriched for the GO-term “RNA modification” and “tRNA processing” (Fig. S2C). Notably, mRNAs that were more soluble upon Not4 depletion were mRNAs that tended to be less soluble in wild-type cells (Fig. 2G).

Taken together, these results indicate that specific features of the mRNAs, or specific mRNA families, indicate how their solubility will be impacted upon depletion of Not1 or Not4.

### Following depletion of the Not proteins changes in solubility and relative degradation correlate

**Fig. 3:**
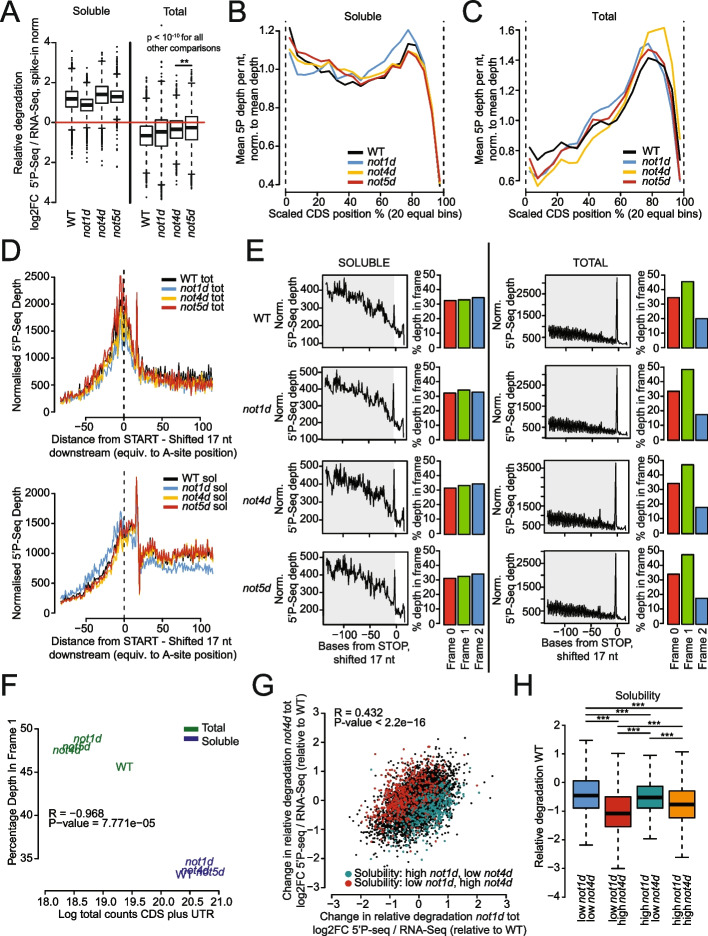
Soluble mRNAs show more relative degradation but less detectable co-translational decay than insoluble mRNAs. **A** Box plot analysis indicating levels of 5′P mRNA decay intermediates compared to total RNA normalized by spike in control RNA in cells before (WT) and after Not1 (*not1d*), Not4 (*not4d*), and Not5 (*not5d*) depletion, in soluble and total RNA pools. Significance of differences is indicated at the top of the box plots—*p*-values are calculated using a two-sided Welch two-sample *t*-test. **B–E** Positions of 5′ ends of decay intermediates were shifted 17nt downstream to simulate the A-site of the adjacent ribosome and create metagene profiles of 5′P mRNA decay intermediates for WT and after Not1, Not4, and Not5 depletion; **B** and **C** over the whole CDS for soluble (**B**) and total (**C**) RNA pools; **D** around the start codon for the total (upper panel) or the soluble (lower panel) RNA pools; **E** before the stop codon in soluble (left) and total (right) RNA pools, with an indication of the percentage of 5′P decay intermediate 5′ ends (no shift) in each of the reading frames over the entire ORFs for the cells before (WT, first row) and after Not1 (*not1d*, second row), Not4 (*not4d*, third row), and Not5 (*not5d*, fourth row) depletion. **F** Scatterplot representing percentage of 5′P decay intermediate 5′ ends in Frame 1 within the soluble and total RNA pools for the indicated strains. **G** Scatterplot comparing relative degradation (corrected to WT) in *not1d* tot and *not4d* total RNAs. Transcripts with solubility high in *not1d* and low in *not4d* are indicated in green, and low in *not1d* and high in *not4d* are red, related to Fig. 2C. **H** Same analysis as Fig. 2D but for relative degradation in wild-type cells

We next determined how the relative degradation of the total and soluble RNA pools was impacted upon depletion of the Not proteins by comparing the fraction of molecules undergoing degradation to the overall mRNA abundance. The overall higher relative degradation in the soluble RNA pool compared to the total RNA pool was maintained following the depletion of the Not proteins (Fig. 3A). However, the relative degradation of the soluble mRNA pool was decreased upon Not1 depletion but increased upon Not4 depletion, and to a lesser extent upon Not5 depletion. For the total RNA pool, the relative degradation slightly increased following the depletion of any of the Not proteins.

The metagene profiles of 5′P decay intermediates for the soluble mRNAs were overall similar before and after Not protein depletion, except for a slight decrease in 5′P decay intermediates in the first half of the CDS and increase in the second half of the CDS upon Not1 depletion (Fig. 3B). For the total RNAs, a decrease in 5′P mRNA ends in the first half of the CDS and an increase in the second half of the CDS was observed upon depletion of each of the Not proteins, most prominently upon Not4 depletion (Fig. 3C).

We looked more closely in the region surrounding the beginning of the CDS. For the total RNA pool, there was a peak of 5′P mRNA ends centered around the start codon (Fig. 3D, upper panel). For the soluble RNAs, a peak at start was so not well pronounced (Fig. 3D, lower panel). In both soluble and total RNAs, a peak at about 20 nucleotides downstream of the start codon was detectable. Notably, a peak of ribosome footprints around this position has been seen in ribosome profiling data sets (e.g., [61]). The metagene profiles around the start were similar before and after depletion of the Not proteins, with the exception of the slightly increased reads upstream of the start and decreased reads downstream of start for the soluble mRNAs upon Not1 depletion that could be indicative of more ribosome pausing at start.

We next focused on the region before the stop codon. In particular, we inspected the profiles for three nucleotide-periodicity expected for 5′P mRNA reads resulting from co-translational decay. Periodicity was well detectable for the total RNAs but not for the soluble RNAs (Fig. 3E). This three nucleotide-periodicity was improved for the total RNA pool upon depletion of each of the Not proteins (Fig. 3F), and this was unrelated to the depth of the sequencing libraries (Fig. S2D). For the total RNA samples, but not for the soluble RNAs, an important peak was detected at the stop codon, suggesting that ribosome recycling might be slow for insoluble mRNAs. Upon depletion of each Not protein, the peak at the stop codon increased in the soluble RNAs and it increased for the total RNAs upon Not4 depletion.

The pool of mRNAs that showed the most extreme change in solubilities upon Not1 and Not4 depletions (Fig. 2C) were distinguishable by opposing behaviors with regard to their relative degradation: those mRNAs more soluble upon Not1 or Not4 depletion tended to have increased relative degradation (Fig. 3G). We also noted that mRNAs that become more soluble upon Not4 depletion and tended to be less soluble in wild-type cells as mentioned above consistently also had less relative degradation in wild-type cells (Fig. 3H). These findings indicate that changes in solubility and relative degradation correlate.

### Metagene profiles of decay intermediates distinguish mRNAs inversely impacted by Not1 and Not4

**Fig. 4:**
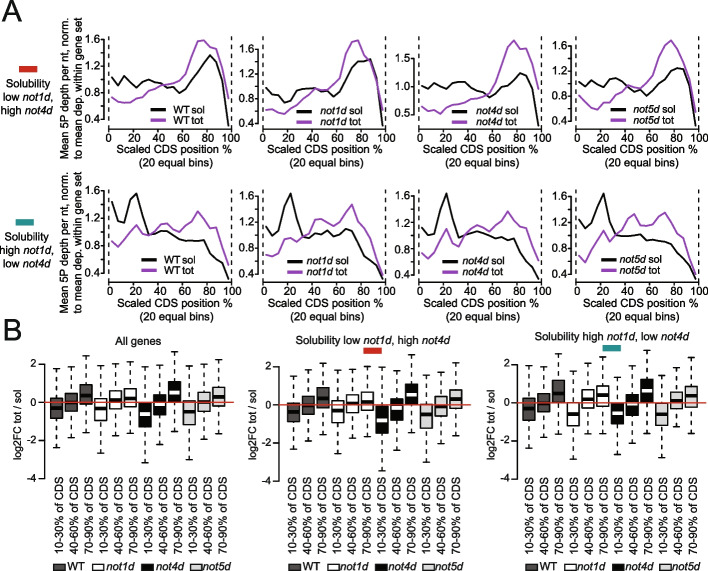
mRNAs with solubilities inversely regulated by Not1 and Not4 show very different co-translational decay patterns. **A** Metagene analysis of 5′P-Seq for total and soluble mRNAs of the red and green categories of Fig. 2C in wild type and after Not1, Not4, or Not5 depletions. **B** Box plot analysis of the 5′P-Seq reads for total versus soluble mRNAs, comparing proportion of reads falling between 10 and 30%, 40 and 60%, and 70 and 90% of CDSs, for all mRNAs (left), mRNAs of the red category (middle), or green category (right). Transcripts are only included in the analysis if their CDS is covered by at least 20 5′P-Seq reads in both soluble and total

We focused on the mRNAs that showed opposite solubility regulations upon Not1 and Not4 depletion (green and red categories of Fig. 2C). The 5′P-Seq metagene profiles for these 2 groups of mRNAs were very different (Fig. 4A and see box plots on Fig. 4B for quantification of reads in the total versus soluble RNA pools for different portions of the CDS). This was most striking for the soluble RNA pool, whereby for the mRNAs of the red category (less soluble upon Not1 depletion) there was a constant lower amount of reads in the first half of the CDS and increased reads in the second half of the CDS. Instead, for the mRNAs of the green category (more soluble upon Not1 depletion), the reads decreased in the second half of the CDS and showed a peak of reads centered at 20% of the CDS. For this latter category, the reads for the total RNA pool were less different between the first and second halves of the CDS than for the first category that showed a pattern more similar to that seen for all mRNAs.

For mRNAs less soluble upon Not1 depletion (red mRNAs) (Fig. 4A, upper panels, and Fig. 4B, middle panel), depletions of Not4 or Not5 decreased 5′P-Seq reads in the total versus soluble RNA pools in the first third (10–30%), whereas Not1 depletion slightly increased 5′P-Seq reads in the total versus soluble RNA pools in the first third (10–30%) and decreased them in the last third (70–90%) of the CDS. For the mRNAs less soluble upon Not4 depletion (green mRNAs) (Fig. 4A, lower panels, and Fig. 4B, right panel), depletions of Not1 or Not5 increased reads in the middle of the CDS (40–70%) for the total RNAs.

These results show that co-translational decay patterns are very different for the two mRNA categories whose solubility is inversely impacted by Not1 or Not4 depletion. They additionally suggest that Not5 works with Not4 for the red mRNA category but with Not1 for the green category.

### A-site RDOs calculated from 5′P Seq data distinguish soluble and total RNA pools and the actions of the different Not proteins

**Fig. 5:**
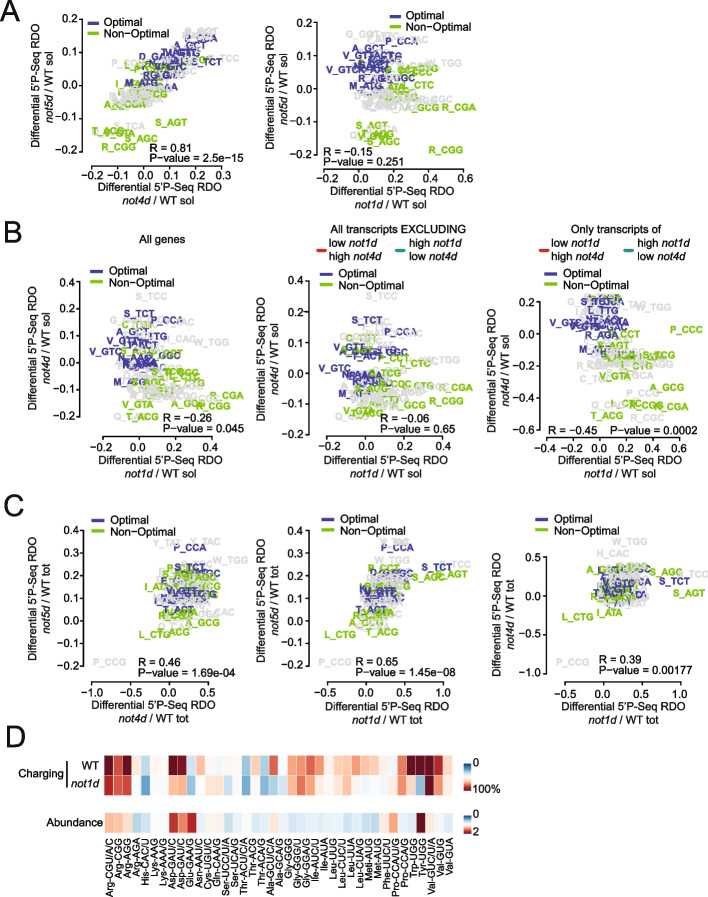
5′P-RDO changes within the soluble RNA pool correlate with codon optimality upon Not4 and Not5 depletion. Scatterplot analyses comparing pairwise changes in 5′P-RDOs relative to WT in: **A** soluble RNAs upon Not4 and Not5 (left) or Not1 and Not5 (right) depletion; **B** soluble RNAs for Not1 and Not4 depletion for all mRNAs (left), all mRNAs excluding mRNAs of the red and green categories of Fig. 2C (middle) or only for mRNAs of the red and green categories (right); **C** for total RNAs upon Not1, Not4, and Not5 depletion. **D** tRNA microarray analysis of aminoacyl-tRNA levels in Not1 depleted and corresponding wild-type strain (top lane) and the relative total tRNA abundance (bottom lane). The abundance was measured relative to the wild-type strain. The arrays are an average of three biological replicates. Confidence intervals between replicate 1 and 2, 1 and 3, and 2 and 3 were 97%, 98%, and 98% for the charging arrays of the Not1 depleted cells; 97%, 97%, and 97% for the abundance arrays of the Not1 depleted cells; 97%, 97%, and 97% for charging arrays of the wild-type; and 95%, 94%, and 98% for the abundance arrays, respectively. tRNA probes are depicted with their cognate codon and the corresponding amino acid

We compared A-site 5′P-RDO changes of the soluble RNA pools following Not1, Not4, or Not5 depletions. In the soluble RNA pool, the A-site 5′P-RDO changes observed upon Not4 and Not5 depletion correlated and they correlated with codon optimality (Fig. 5A, left panel). Instead, the A-site 5′P-RDO changes observed upon Not1 and Not5 depletion showed a minor anti-correlation (Fig. 5A, right panel). Such an inverse correlation was more pronounced and significant for the A-site 5′P-RDO changes observed upon Not1 and Not4 depletion (Fig. 5B, left panel). It was abolished if the mRNAs of the red and green categories were removed (Fig. 5B, middle panel) and was instead more important and much more significant when only the mRNAs of the red and green categories were analyzed (Fig. 5B, right panel), with a clear codon-optimality related effect. It is interesting to note that RDO changes in cells lacking Not5 compared to wild-type cells measured by Ribo-Seq previously and shown to correlate with 5′P-RDO changes upon Not1 depletion [17] showed instead a mild inverse correlation with 5′P-RDO changes upon Not5 depletion (Fig. S3A). This suggests that in *not5Δ* cells limiting amounts of Not1 have a dominant impact on RDOs.

For the total RNA pool, the 5′P-RDO changes observed immediately upon depletion of the Not proteins correlated, most importantly for Not1 and Not5 depletions and least importantly for Not1 and Not4 depletions, with some specific codons, e.g., Leu (CTG) and Pro (CCG) striking out (Fig. 5C). 5′P-RDO changes in the total and soluble RNA pools after depletion of each respective Not protein did not show any significant correlation (Fig. S3B).

5′P-RDO increases at Ser codons were most dramatic upon Not1 depletion (Fig. 5C). A change of metabolic flow from serine to alanine is known to occur during anoxia. Hence, depletion of Not1 may have immediate changes on metabolism, which could be directly detected by the serine levels and tRNA charging, and would explain the dramatic 5′P-RDOs increases at Ser codons upon Not1 depletion. To test this, we determined the relative changes in the total RNA levels and the percentage of charged tRNA for each isoacceptor, before and following Not1 depletion. We observed a slight but insignificant decrease of the tRNA^Ser^ levels following Not1 depletion, but the levels of two seryl-tRNA^Ser^ isoacceptors markedly decreased (Fig. 5D).

### mRNAs whose overexpression and solubility are inversely impacted by Not1 or Not4 depletion show relatively lower Not4 cross-linking

**Fig. 6:**
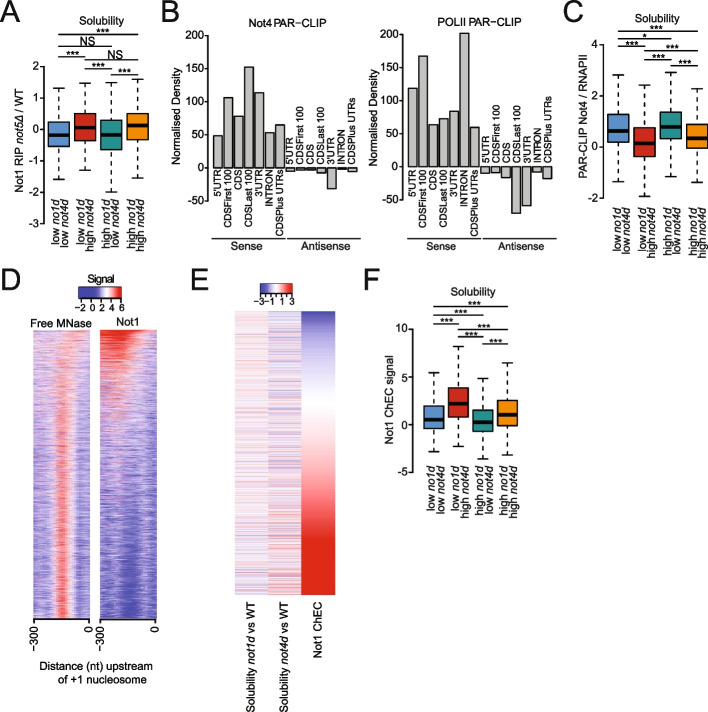
Not4 cross-linking correlates with changes in mRNA solubilities upon Not4 depletion and Not1 promoter association correlates with mRNAs less soluble upon Not1 depletion.** A** Box plot comparing Not1 RIP in cells lacking Not5 for the categories of mRNAs color-coded in Fig. 2C. Significance of differences is indicated at the top of the box plots—*p*-values are calculated using a two-sided Welch two-sample *t*-test. **B** Comparative cross-linking (normalized density) of Not4 (left) and RNAPII (right) to different mRNA sequences as indicated. **C** Same as panel A, but comparing the relative Not4/RNAPII cross-linking to the different classes of mRNAs. **D** Heat map comparing signal of free MNAse and Not1-MNase DNA cleavage events at promoter regions (ChEC). **E** Heat map comparing Not1-ChEC signal and changes in mRNA solubilities upon Not1 and Not4 depletion. **F** Box plot analysis of the Not1-ChEC signal in the different promoters of the genes encoding the different classes of mRNAs color-coded in Fig. 2C. Significance of differences is indicated at the top of the box plots—p-values are calculated using a two-sided Welch two-sample *t*-test

The inverse impacts of Not1 and Not4 on mRNA solubilities and 5′P-RDOs raise the question of the mechanism, and as to whether the regulation occurs via their mRNA binding. In previous work, we defined Not1 mRNA binding by RNA immunoprecipitation (RIP) in wild-type cells and in cells lacking Not5. We noted an interesting significantly higher Not1 RIP in *not5Δ* for the mRNAs more soluble upon Not1 depletion (Fig. 6A). This was not related to the overall increased size of the mRNAs bound by Not1 in the absence of Not5 (Fig. 2F).

We thus focused on Not4 whose association with RNAs has not yet been characterized. To define to which mRNAs Not4 binds in vivo, we used a photoactivatable ribonucleoside-enhanced crosslinking and immunoprecipitation (PAR-CLIP) approach [62]. In two independent biological replicates, we identified the positions of Not4 cross-linking to mRNAs, resulting in T to C transitions (Table S3). The replicates showed high correlation (Fig. S4A). The reads were distributed throughout transcribed sequences, mapping to coding sequences (CDSs), 3′ and 5′ untranslated regions (UTRs), and introns, of the sense and anti-sense regions (Fig. 6B, left). Overall reads correlated with T to C transitions on coding sequences as well as on introns (Fig. S4B). There was a good correlation between cross-linking of Not4 and cross-linking of RNA polymerase II (RNAPII) [63] (Fig. 6B, right) on all sense (Fig. S4C), and anti-sense (Figure S4D) sequences, suggesting Not4 binds mRNA ubiquitously during transcription. Despite the global correlation, we noticed some differences. For instance, RNAPII cross-linked more efficiently to introns and less to CDSs, whereas Not4 was cross-linked better to 3′ UTRs (Fig. 6B), and the correlation was better for sense than for anti-sense regions (Fig. S4C and D). Moreover, the patterns of Not4 and RNAPII cross-linking differed along mRNAs, with RNAPII cross-linking more prominent in 5′UTRs and at the very beginning of CDSs, but Not4 cross-linking instead more prominent on CDSs, most striking at the end of coding sequences and also more prominent in 3’UTRs (Fig. S4E). Only few mRNAs (270) had at least twofold less cross-linking of Not4 than expected from the global correlation between Not4 and RNAPII cross-linking whereas 1222 mRNAs had more than twofold higher cross-linking of Not4 than expected (Fig. S4F, in purple). GO-term analysis of this latter mRNA group revealed enriched categories, including “mitochondrion organization,” “response to oxidative stress,” “proteolysis involved in cellular protein catabolic processes,” “protein complex biogenesis,” “protein folding,” and “cytoplasmic translation” (Fig. S4G), that have functionally been connected to Not4 in previous studies [17, 42, 49, 53, 64, 65].

We compared the mRNAs cross-linked to Not4 to the sets of mRNAs differentially solubilized described above (Fig. 2C). mRNAs less soluble upon Not4 depletion showed higher Not4 cross-linking (Fig. 6C, blue and green mRNAs), suggesting that Not4 mRNA binding plays a role in solubility of these mRNAs. Notably, this trend was also detectable, albeit to a lesser degree when Not4 cross-linking rather than relative Not4 to RNAPII cross-linking was considered (Fig. S4H). Instead, it was not as significant for all comparisons when RNAPII (Fig. S4I) or expression levels (RNA-Seq) (Fig. S4J) were considered. This indicates that it is not only related to Not4 cross-linking to mRNAs according to expression levels, but to functions of Not4 after transcription.

### Not1 binds promoters broadly but with specificity related to mRNA solubility

Previous work has shown that Not5-dependent Not1 binding to ribosomal protein (RP) mRNAs occurs during transcription and regulates their translation in a manner dependent upon Not5 [48]. Above we showed that the mRNAs more soluble upon Not1 depletion have higher Not1 RIP in *not5Δ*. We thus mapped Not1 promoter binding genome-wide to determine whether Not1 association with promoters correlated with the Not1-dependent fate of mRNAs in the cytoplasm, in particular their solubility. We fused Not1 to the micrococcal nuclease (MNase) at its own genomic locus, monitored chromatin cleavage events (ChEC), and compared them to the cleavage pattern by the free MNase. In total, 4923 promoters showed cleavages by Not1-MNase above the threshold of significance, and the pattern of Not1-MNase was specific compared to the pattern obtained with free MNase (Table S4 and Fig. 6D). Higher binding of Not1 at promoters correlated with lower solubility of mRNAs upon Not1 depletion and higher solubility of mRNAs upon Not4 depletion (Fig. 6E). In particular, the promoters driving transcription of mRNAs showing lower solubility upon Not1 depletion but higher following Not4 depletion (red mRNAs) showed higher Not1 promoter binding (Fig. 6F). These results suggest that the cytoplasmic fates of mRNAs defined by Not1 and Not4 in opposing manner are likely set in the nucleus during transcription by Not1 promoter binding and resulting lower Not4 mRNA cross-linking.

## Discussion

### Solubility as a mechanism by which Not proteins regulate co-translation dynamics

Several recent studies provide evidence that the Not proteins are key for codon-optimality-related changes in mRNA stability [50, 66]. A beautiful structure of Not5 associated with the translating ribosome corroborates the idea that the Ccr4-Not complex can monitor codon optimality, but a mechanism linking this ribosome docking to control of mRNA decay is elusive. Our work has indicated that ribosome dwelling occupancy evaluated by ribosome profiling (i.e., valid for the soluble mRNA pool) is regulated according to codon-optimality by the Not subunits of the Ccr4-Not complex. We proposed that this could occur via the dynamic formation and dissolution of Not condensates during translation [17]. In this current study, we tested the model further by comparing the soluble mRNA pool to the total mRNA pool that includes all insoluble mRNAs. We find that soluble mRNAs differ from the insoluble mRNAs by higher relative degradation and lower content in ribosome dwelling at non-optimal codons, and that Not1 and Not4, both acting with Not5, inversely modulate solubility for different mRNAs. These findings indicate that modulation of solubility is the mechanism by which Not5-ribosome binding can regulate mRNA turnover and translation dynamics according to codon optimality.

### Soluble and insoluble RNA pools have distinguishable co-translational dynamics

We show that the solubility of mRNAs is a determining factor in regulating co-translation dynamics. Indeed, soluble mRNAs show more relative degradation but less detectable co-translational decay compared to insoluble mRNAs, namely all mRNAs that are not soluble without distinction of the different types of insoluble mRNAs. In soluble fractions ribosome movement is faster than the action of the 5′-3′ exonuclease activity. Alternatively, or in addition, mechanisms other than co-translational decay might generate decay intermediates. These could be No-Go-Decay (NGD) that can generate 5′ to 3′ decay intermediates upstream of collided ribosomes [67], and more importantly post-translational decay that is not marked by ribosome dwelling.

Soluble and insoluble mRNAs also exhibit a different distribution of 5′P decay intermediates along CDSs most likely due to differences in ribosome dwelling and hence velocity. Indeed, we show that ribosomes dwelling at non-optimal codons are more prominent in the insoluble RNA pool. Alternatively, the delay of 5′ to 3′ decay initiation after the last trailing ribosome, indicated by higher 5′P-RDOs at non-optimal codons for the second half of CDSs compared to the first half, is less significant for soluble mRNAs, since RNA-Seq reads relative to 5′P-Seq reads in the first half compared to the second half of CDSs are higher.

### mRNA solubility is inversely regulated by Not1 and Not4, both working with Not5

In previous work, we proposed that tethering of mRNAs by Not proteins to condensates, thus to insoluble RNA pools, in particular at start and at non-optimal codons, modulates translation elongation dynamics [17]. In line with this model, and based on the results discussed here, depletions of the Not proteins increase overall the relative degradation and, in addition, three-nucleotide periodicity overall. This implies a more prominent role of the Not proteins for general decay than for co-translational decay. It is consistent with Not proteins being subunits of the major eukaryotic deadenylase complex [22] and with the role of Not4 for a bypass quality control pathway [68].

**Fig. 7:**
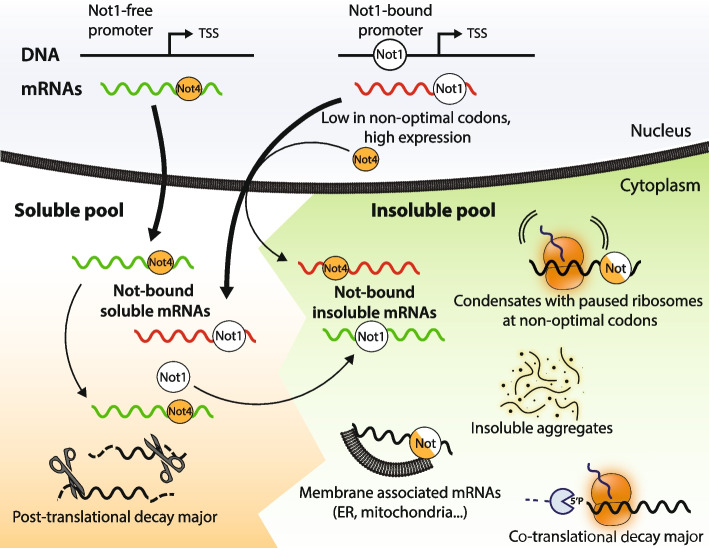
Model for the opposing regulation of mRNA solubility by Not1 and Not4. mRNAs exhibit diverse solubilities, and mRNAs that are not soluble can be either in functional condensates, associated with membranes, or in cytosolic aggregates. Not1 and Not4 have opposing effects on mRNA solubility and solubility of some mRNAs (designated red) is promoted by Not1, whereas for another set (green), solubility is promoted by Not4. mRNAs that are less soluble upon Not1 depletion but more upon Not4 depletion (red) are transcribed from genes with high Not1 recruited at their promoters. Not4, also co-transcriptionally recruited to mRNAs, opposes their solubilization. These mRNAs have low non-optimal codon content and are highly expressed. They show high Not1 binding in *not5Δ* (RIP) background and Not4 cross-linking is lower compared to the mRNAs that instead are less soluble upon Not4 depletion but more upon Not1 depletion (green mRNAs). These latter mRNAs are generally more soluble and they are less likely to have co-transcriptional recruitment of Not1. They remain soluble in a Not4-dependent manner. Solubility of these mRNAs is compromised by the depletion of Not4, possibly because Not1 recruitment is thereby enabled, since depletion of Not1 instead increases their solubility. Note that Not1 and Not4 are placed on mRNAs to indicate functional interaction, but do not infer direct mRNA binding

While these findings indicate that the Not proteins act together and in a similar manner, several observations contradict this simple interpretation. First, depletion of Not4 or Not5 results in higher, but that of Not1 lower, relative degradation for soluble mRNAs. Higher levels of 5′P decay intermediates in the second CDS half are observed most significantly upon depletion of Not1 for soluble mRNAs, but upon depletion of Not4 for total mRNAs. Importantly, mRNA solubility was mildly altered by depletion of Not5, but inversely impacted by the depletions of Not1 and Not4 (see model in Fig. 7). In particular, mRNAs more soluble upon Not4 depletion (red category) have a relatively lower content in non-optimal codons and the 5′P-RDO changes for soluble mRNAs that correlate upon Not4 and Not5 depletion and correlate with codon optimality can be related to the mRNAs with low non-optimal codons becoming soluble. Instead, they become insoluble upon Not1 depletion; hence, 5′P-RDO changes tend to be inverse.

In contrast to Not1 and Not4 depletion, Not5 depletion showed only minor effects on mRNA solubility. We expect that Not5 association with ribosomes is key for regulations by Not1 and Not4 since recent work features a key role of Not5 in monitoring codon optimality via its binding to post-translocation ribosomes [50]. Indeed, Not5 works together with Not1 for mRNAs solubilized upon Not1 depletion (green category) and 5′P-RDO changes for total RNAs correlate best upon Not1 and Not5 depletion, but, as mentioned above, with Not4 for mRNAs solubilized upon Not4 depletion (red category). Thus, Not5 works with both Not1 and Not4 that have opposing roles, and these opposite effects most likely cancel each other upon Not5 depletion.

### Targets of Not1 regulation are conserved

A recent study has investigated the global effects of CNOT1 knockdown in human cell lines [69]. An interesting parallel can be seen between the classes of mRNAs regulated in human by CNOT1, either at the mRNA stability level or at the translation efficiency level, and those regulated by Not1 in yeast. Indeed, in yeast, shorter mRNAs are less soluble upon Not1 depletion. Such mRNAs would be expected to be more stable in Not1 knockdown and this correlates with the finding that in CNOT1 knockdown half-life of shorter mRNAs increases. In yeast, mRNAs less soluble upon Not1 knockdown are enriched for mRNAs translated at the ER. In human cells, upon CNOT1 depletion, ER-targeted mRNAs were enriched within mRNAs with reduced translation efficiency, calculated as a ratio of ribosome footprints determined by ribosome profiling relative to total RNA. Ribosome footprinting can only evaluate soluble RNA fractions; thus if these mRNAs are less soluble upon CNOT1 depletion, one would expect a drop in translation efficiency. In an opposite manner, in yeast, Not1 depletion increases the solubility of mitochondrial mRNAs and CNOT1 knockdown increased their translation efficiency in human cells, a phenotype that would be observed if the mRNAs are more soluble. These comparisons indicate that inherent mRNA characteristics of some gene groups regulated by Not1 are conserved from yeast to human.

### Regulation of mRNA solubility is set during transcription

It might appear counter-intuitive that two subunits of the same complex have opposing roles. However, having two opposing factors working in the context of a single multi-subunit complex might be essential to fine-tune co-translational dynamics. It should also be noted that in human cells CNOT4 is not a stable subunit of the Ccr4-Not complex. It raises the question as to how the opposing roles of Not1 and Not4 are set. The inverse regulation by Not1 and Not4 is importantly correlated with levels of Not1 promoter binding and Not4 cross-linking, and our data clearly indicates that Not4 association with mRNAs occurs during transcription. Hence, mRNA solubility appears to be set already during transcription. It could be that high Not1 promoter binding can result in higher Not1 association with newly produced mRNAs and thus lower Not4 cross-linking, whereas if less Not1 is present at the promoter, higher Not4 association with newly produced mRNAs can occur (see model on Fig. 7). After translation onset, the roles of Not1 and Not4 may dynamically interchange during translation elongation, in line with our previous finding that Not condensates are dynamic [17]. This might be mediated by Not1 and Not4 association with ribosome-interacting Not5. Not condensates may be more or less insoluble according to their complexity or their association for instance with membranes.

Not1 appears to be important for solubility of highly expressed mRNAs and important for cytoplasmic translation, not only of ribosomal protein mRNAs, whose solubilities are inversely regulated by Not4, but also of mRNAs encoding tRNA processing and rRNA modification enzymes that are important for the production of a functional translation machinery. It could be that Not1 plays a role to prevent aggregation or condensation of such mRNAs during translation and/or to release such mRNAs from condensates. Indeed, a role of Not1 to solubilize Dhh1 condensates has been demonstrated [70]. Instead, mRNAs with higher Not4 cross-linking and lower Not1 at promoters tend to be more soluble (blue and green categories) suggesting that higher association of Not4 with mRNAs counteracts a more general effect of Not1 to contribute to mRNA insolubility. Depletion of Not1 and Not4 also have similar effects, rendering shorter mRNAs less soluble but solubilizing longer mRNAs. Length of mRNAs may counterbalance the effect of Not proteins co-transcriptionally recruited to mRNAs by co-translational recruitment of additional factors.

## Conclusions

Our results reveal that mRNA solubility defines dynamics of co-translation events and is oppositely regulated by Not1 and Not4, a mechanism that we additionally determine may already be set by Not1 promoter association in the nucleus. From our results, one might additionally consider that the Not1 promoter association has an impact on how much Not4 can become associated with the mRNA transcribed from that promoter. In addition, we demonstrate a correlation between changes in codon-optimality-related co-translation decay and solubility of mRNAs. We believe that these findings may open the door to understanding the mechanism for turnover of mRNAs according to codon-optimality.

## Materials and methods

### Strains, plasmids, and culture conditions

Plasmids and strains are listed in Table S5. The Not4 and Not5 degron strains (13771 and 13772) were created in strain MY13472 (*MAT*α *leu2-3,112 his3,15 ura3-1::pADH1-OsTIR1-URA3 ade2 trp1-1 can1-100*) and PCR amplification of a 9Myc-NATMX4 cassette with Not4- and Not5-specific primers using plasmid pE641. The Not1 degron strain (13517) has already been described [17]. The strains were verified by PCR. Not protein depletions were obtained by addition of auxin (3-indoleacetic acid, Sigma-Aldrich I2886, stock solution at 250 mM in EtOH) at 1 mM final for 15 min to exponentially growing cells diluted to OD600 0.3 after an overnight culture in glucose-rich medium (YPD), when they reached OD600 0.8. Equivalent amounts of EtOH 100% were added for the control. All experiments were performed with cells growing in YPD. The strain expressing the Not4-HTB fusion (MY11050) was generated in BY4741 (*MATa leu2Δ20 ura3Δ met15Δ his3Δ1*) with Not4-specific primers by PCR using pE557, the strains expressing the Not1-MNase fusion (MY12601) was generated with Not1-specific primers by PCR using pE611 respectively. The free MNase strain was MY13783 (*MATa tor1-1 fpr1::loxP-LEU2-loxP RPL13A-FKBP12::loxP ura3::MNAse-URA3*).

### RNA preparation

Total RNA was prepared either by the hot acid phenol method [71] or cells were prepared and lysed as for polysome profiling [42] and the soluble RNA pool was prepared from the lysate. Briefly, for total RNA, cell pellets from 30 ml of exponentially growing yeast were resuspended with 400 μl of TES buffer (10 mM Tris HCl pH 7.5, 10 mM EDTA and 0.5% SDS) to which 400 μl of acid phenol were added. After vortexing and incubation for 10 min at 65 °C, RNA was extracted. For soluble RNA, cell pellets from 30 ml of exponentially growing yeast were resuspended in 400 μl of lysis buffer (20 mM Hepes 20 mM KCl, 10 mM MgCl2 1% triton, 1 mM PMSF, 1 mM DTT supplemented with a cocktail of protease inhibitors and with 0.1 mg/ml of cycloheximide) to which 200 μl of glass beads was added. Cells were vortexed at 4 °C for 15 min and spun at 15,000 K for 1 min. The supernatant was transferred to a new tube and spun a further 20 min at 4 °C. The supernatant was combined with 400 μl of acid phenol for further RNA extraction as for the total RNA pool. For normalization, we spiked in each RNA sample, whether soluble or total, a constant amount of RNA from *Schizosaccharomyces pombe*, before the RNA samples were then split in 2, one aliquot subjected to RNA-Seq to determine the transcriptome and the other to 5′P-Seq to determine mRNA decay.

### Protein extraction and analysis

Total protein was prepared by post-alkaline lysis and analyzed by western blotting with antibodies to Not1, Not4, and Not5 that were our own polyclonal antibodies previously described [72, 73]. Antibodies to Myc were commercial (Sigma M5546).

*5*′*P-Seq.*

RNA was prepared from 50 ml of exponentially growing cells in YPD and treated or not with auxin. HT-5PSeq libraries were generated as reported [74] with minor modifications. In brief, 15 μg RNA, containing 5% total RNA from *Schizosaccharomyces pombe* as spike-in, was used. Each sample was split in two. One part was used for preparing conventional HT-5PSeq libraries and the other part was random fragmented prior to the preparation of HT-5PSeq libraries (negative control).

For HT-5PSeq Libraries: 7.5 μg RNA was ligated over night at 16 °C to r5P_RNA_MPX oligo (CrArCrGrArCrGrCrUrCrUrUrCrCrGrArUrCrU rXrXrXrXrXrX rNrNrNrNrNrNrNrN) carrying a sample barcode (rX) and unique molecular identifiers (rN). Ligase was deactivated using 5 mM EDTA and heat at 65 °C for 10 min (up to X individual barcoded RNA ligations were pooled) and subsequent purified using 1.8 × volumes of RNAClean XP beads (Beckman Coulter). Ligated RNA was then reverse transcribed using random hexamer (5Pseq-RT, GTGACTGGAGTTCAGACGTGTGCTCTTCCGATCTNNNNNN, 20 μM) and oligo-dT (5Pseq-dT, GTGACTGGAGTTCAGACGTGTGCTCTTCCGATCTTTTTTTTTTT at 0.05 μM) oligos to prime. After, the remaining RNA was degraded using NaOH. Ribosomal RNA was removed using previously described rRNA DNA oligo depletion mixes, following a duplex-specific nuclease (DSN, Evrogen) digestion. rRNA-depleted cDNA was amplified by PCR (17 cycles) and final product was enriched for fragments with the range of 300–500 nt using Ampure XP.

Size selected HT-5P Libraries were quantified by fluorescence (Qubit, Thermo Fisher), size estimated using an Agilent Bioanalyzer and sequenced using a NextSeq500 Illumina sequencer (75 cycles High output kit).

### Not4 PAR-CLIP

Cells expressing Not4 tagged at the C-terminus with the HTB tag [75] from its endogenous locus and endogenous promoter were grown in duplicates in the presence of 4-thiouracil and then UV-irradiated at 365 nm to cross-link proteins with RNA. Not4 was purified under denaturing conditions and libraries were prepared from the co-purified RNA and sent for deep sequencing as described [63].

### Chromatin endogenous cleavage (ChEC-Seq)

Not1 ChEC-Seq experiments were essentially performed as previously described [76] with the following modifications. Cells in which MNase was fused at the C-terminus of the endogenous *NOT1* or *NOT5* genes generated using PCR-amplification of tagged alleles and homologous recombination according to standard techniques [77] were used to determine Not1 and Not5 binding. Cells in which MNase was placed under the control of *REB1* promoter were used as a control [77]. One sample corresponds to 12 ml of culture at OD_600_ = 0.7. Cells were washed twice with buffer A (15 mM Tris 7.5, 80 mM KCl, 0.1 mM EGTA, 0.2 mM spermine, 0.5 mM spermidine, 1xRoche EDTA-free mini protease inhibitors, 1 mM PMSF) and resuspended in 200 μl of buffer A with 0.1% digitonin. The cells were incubated for 5 min at 30 °C. Then, MNase action was induced by the addition of 5 mM CaCl2 and stopped at 150 s for Not1 and Not5 ChEC-seq and 20 min for Mnase under the control of REB1 promoter by adding EGTA to a final concentration of 50 mM. DNA was purified using MasterPure Yeast DNA purification Kit (Epicentre) according to the manufacturer’s instruction. Large DNA fragments were removed by a 5-min incubation with 2.5 × volume of AMPure beads (Agencourt) after which the supernatant was kept, and MNase-digested DNA was precipitated using isopropanol. Libraries were prepared using NEBNext kit (New England Biolabs) according to the manufacturer’s instructions. Before the PCR amplification of the libraries small DNA fragments were selected by a 5-min incubation with 0.9 × volume of the AMPure beads after which the supernatant was kept and incubated with the same volume of beads as before for another 5 min. After washing the beads with 80% ethanol the DNA was eluted with 0.1 × TE and PCR was performed. Adaptor dimers were removed by a 5-min incubation with 0.8 × volume of the AMPure beads after which the supernatant was kept and incubated with 0.3 × volume of the beads. The beads were then washed twice with 80% ethanol and DNA was eluted using 0.1 × TE. The quality of the libraries was verified by running an aliquot on a 2% agarose gel. Libraries were sequenced using a HiSeq 2500 machine in single-end mode. To analyze the Not1- and Not5-MNase binding pattern, read ends were considered to be MNase cuts and were mapped to the genome (sacCer3 assembly) using HTSstation (David et al. 2014).

### tRNA microarrays

To determine the fraction of aminoacyl-tRNAs, we followed the procedure described in [78]. For this, total RNA was isolated in mild acidic conditions (pH 4.5) which preserve the aminoacyl-moiety. Each sample was split into two aliquots and one was oxidized with periodate to which the charged tRNAs remain intact and following subsequent deacylation (100 mM Tris (pH 9.0) at 37 °C for 45 min) was hybridized to Cy3-labeled RNA/DNA stem-loop oligonucleotide. The second aliquot was deacylated to receive the total tRNA and hybridized to Atto647-labeled RNA/DNA stem-loop oligonucleotide. Both aliquots were analyzed on the same tRNA microarrays and the ratio of the Cy3 to Atto647 signal provides the fraction of aminoacyl-tRNA for each isoacceptor.

For tRNA abundance, total RNA was isolated at alkaline pH to simultaneously deacylate all tRNAs. tRNAs isolated from Not1 depleted cells were labeled with Cy3-labeled RNA/DNA stem-loop oligonucleotide and were hybridized on the same microarray with tRNAs isolated from the wild-type strain and labeled with Att647-labeled RNA/DNA stem-loop oligonucleotide. The arrays were normalized to spike-in standards, processed, and quantified with in-house python scripts.

### Bioinformatic analyses

#### 5′P-Seq and RNA-Seq

Sequencing files were demultiplexed using bcl2fastq v2.20.0.422 (one mismatch, minimum length 35 nt), and adapters were trimmed using cutadapt 2.3. [79] at default settings, allowing one mismatch and minimum read length of 35nt. In addition to standard illumine dual index (i5, i7), the inline sample and UMI barcode was analyzed using Umitools. Reads were mapped to the concatenated genome of *S. cerevisiae* (R64-1–1) and *S. pombe* (ASM294v2) using STAR.

Second read enables to split reads between oligo-dT or random primer. That information was not used in the current analysis. CDS positions were defined with Ensembl gff version 94 for *S. cerevisiae* (R64-1–1). Counts in *S. cerevisiae* were calculated by aggregating RNA-Seq reads and 5′P-Seq 5′-ends, overlapping CDS positions. Differential expression was performed using DESeq2 [80].

### Solubility

We define this as the log fold change produced by DESeq2, dividing RNA-Seq counts for the soluble fraction in a given sample by the corresponding counts for the total fraction of the same sample.

### Relative degradation

Spike-in *S. pombe* data was used in the calculation of relative degradation. We define it as the log fold change produced by DESeq2, comparing counts in 5′P-Seq to their corresponding RNA-Seq sample using estimateSizeFactors on counts mapping to *S. pombe* to adjust for spike-in. Enrichment is calculated using a hypergeometric test for over-representation of hits in defined gene set for GO SLIM categories for *S. cerevisiae*.

To calculate 5′P-Seq pausing scores, equivalent to A site ribosome dwelling occupancy, the mean depth was calculated 17nt upstream of each codon type for each strain in the regions or transcripts of interest. In all cases, the values are normalized to the mean depth over all codons for the regions or transcripts included in the calculation. Where two conditions are compared, the differential RDO is calculated as the log2 fold change of these normalized values for each codon.

For 5′P-Seq, metagenes at start and stop are calculated by aggregating the depth of 5′ ends at each position relative to start or stop for every CDS and normalizing each by the total depth per million genome-wide. These values are shifted 17nt downstream for equivalency with the A-site position, in the case of co-translational decay.

For scaled metagenes, every CDS was split into 100 equal bins and the mean depth of 5′ ends of RNA-Seq and 5′P-Seq was calculated for each bin. This was averaged over all transcripts of interest and normalized to the mean depth over all nucleotides in this transcript group.

### PAR-CLIP analyses

FASTQ files were adapter stripped, using cutadapt (parameters: -a AGATCGGAAGAGCACACGTCTGAACTCCAGTC –minimum-length = 13 –quality-cutoff = 2) and then mapped using bowtie [81] to sacCer3 (parameters: -v 2 -m 10 –best –strata). High confidence T to C transitions in the cDNA sequence defining the sites of cross-linked 4-thiouracil residues were identified using wavClusteR [82]. These were then normalized to the rate of T bases for the regions of interest to give a normalized density value for cross-linking.

### ChEC-seq analyses

FASTQ files were adapter stripped, using cutadapt (parameters: -a GATCGGAAGAGCACACGTCTGAACTCCAGTCA –minimum-length = 20 –quality-cutoff = 2) and then mapped using bowtie2 [83] to sacCer3 (parameters: -v 2 -m 10 –best –strata). Positions of the + 1 nucleosome associated with each gene were taken from the Saccharomyces Genome Database and read counts overlapping the promoter binding region 400 bp upstream and 100 bp downstream of these position were calculated and normalized to RPKMs. To find the Not1 ChEC signal, the log2 fold change (LFC) of the promoter binding region RPKMs were taken over free MNase. These values were mode-centered to zero (the mode of the LFCs was estimated by fitting a log-normal distribution using “fitdistr” from the R package MASS).

### Ribo-Seq

Values for Ribo-Seq RPKMs were calculated as in our previous paper [17].

### Statistical tests

Reported correlations were the Pearson’s product-moment correlation coefficient and were used as test statistics to generate the associated *p*-value by *t*-test. All correlations and correlation tests were performed on groups of at least 30 in size. Enrichment of gene sets is defined via FDR after Benjamini–Hochberg adjustment from *p*-values generated using a hypergeometric test. All *t*-tests were performed on sample sizes of 30 or higher, where the central limit theorem applies regarding the normality assumption. We use Welch’s *t*-test in all cases, rather than Student’s *t*-test, resulting in more conservative *p*-value, which is more reliable where variances and sample sizes are unequal. In one case, we used a Wilcoxon rank sum test [84] to compare two samples as a non-parametric proxy for a *t*-test, so as to avoid any possible breach of the normality distribution assumption, since both samples had a size of 15 (comparing WT RDOs total/soluble for optimal and non-optimal groups).

## Supplementary Information

**Additional file 1:** Additional figures and figure legends in a single pdf file with: **Figure S1.** Comparison of mRNAs that are more or less soluble. **Figure S2.** mRNAs that are more or less soluble upon Not1 and Not4 depletion are enriched for different GO-terms. **Figure S3.** Comparison of RDO changes between mutants and RNA pools. **Figure S4.** Quality control of the Not4 PAR-CLIP. **Table S5.** List of strains and plasmids.

**Additional file 2:** **Table S1.** 5’P-Seq and RNA-Seq of soluble and total RNA pools before and after depletion of Not1, Not4 and Not5. 

**Additional file 3:** **Table S2.** Changes in solubility upon Not1, Not4 and Not5 depletion.

**Additional file 4:** **Table S3.** Not4 and RNAPII PAR-CLIP. 

**Additional file 5:** **Table S4.** Not1-ChEC-Seq.

**Additional file 6.** A separate pdf file entitled: Uncropped blots. Related to Figure S2A.

**Additional file 7.** Review history.

## Data Availability

tRNA microarray data are accessible under the accession number GSE190658 in the Gene Expression Omnibus (GEO) database [85], 5′P-Seq data under GSE193912 [86]. All code used in the manuscript is available at https://github.com/georgeallenunige/NotTranslation [87], released under an OSI compliant GNU General Public License version 2 (GPL-2.0) with DOI https://zenodo.org/badge/latestdoi/209279257.
