# Supplementary figures and images for: Not1 and Not4 inversely determine mRNA solubility that sets the dynamics of co-translational events
Source: Genome Biol. 2023 Feb 20;24:30. doi: 10.1186/s13059-023-02871-7 (PMC9940351; doi:10.1186/s13059-023-02871-7)

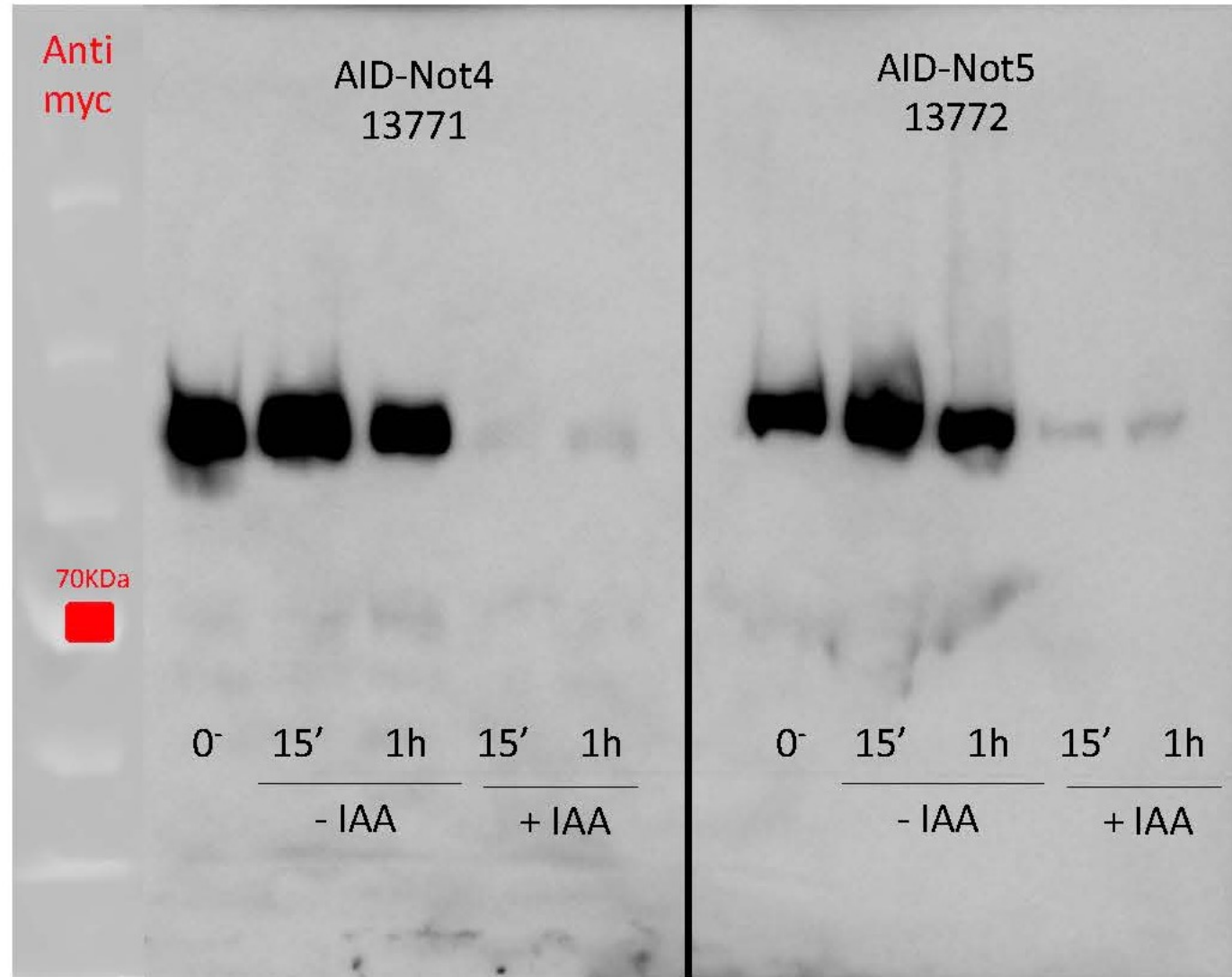

Not1 in Not4-AID

Not1 in Not5-AID

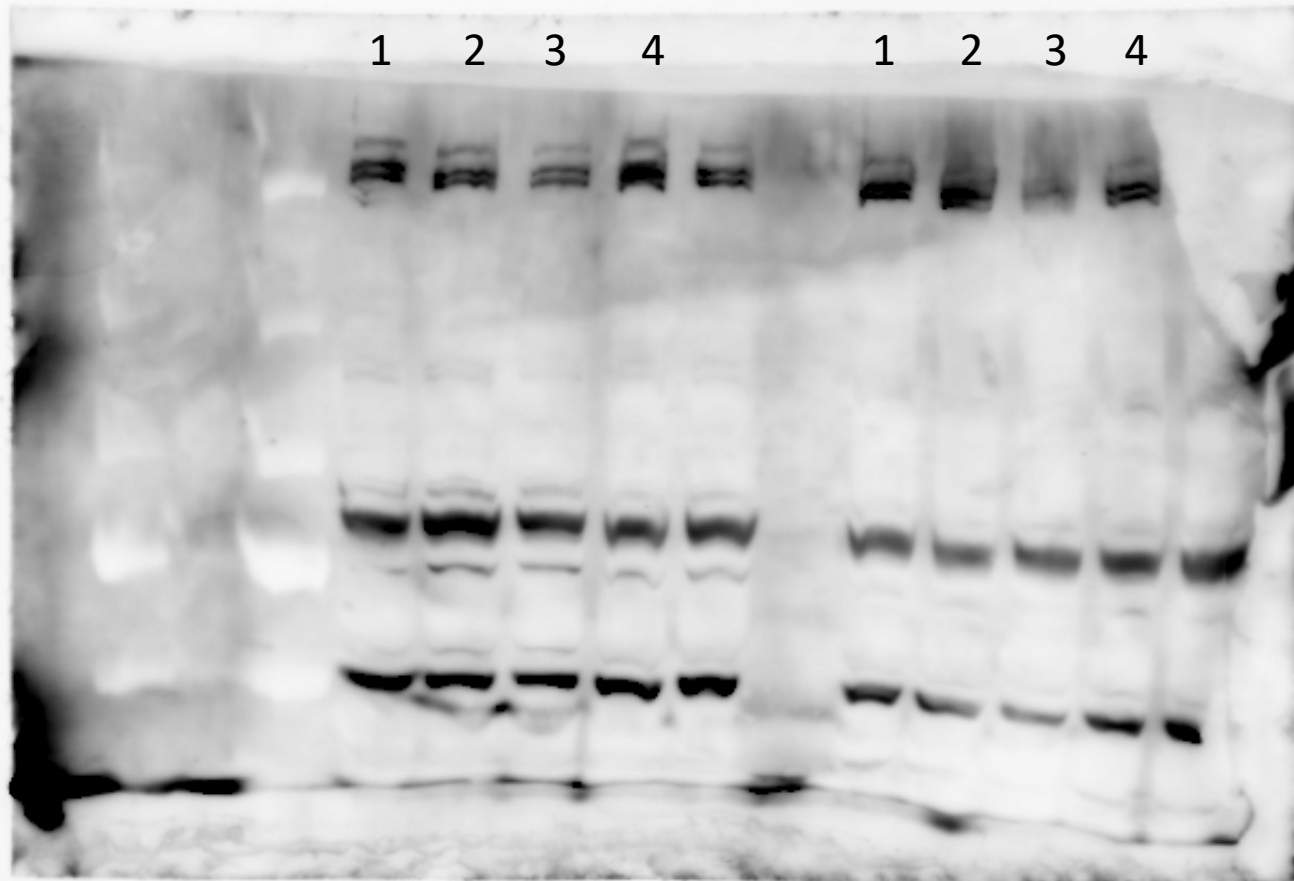

Not4 in Not5-AID

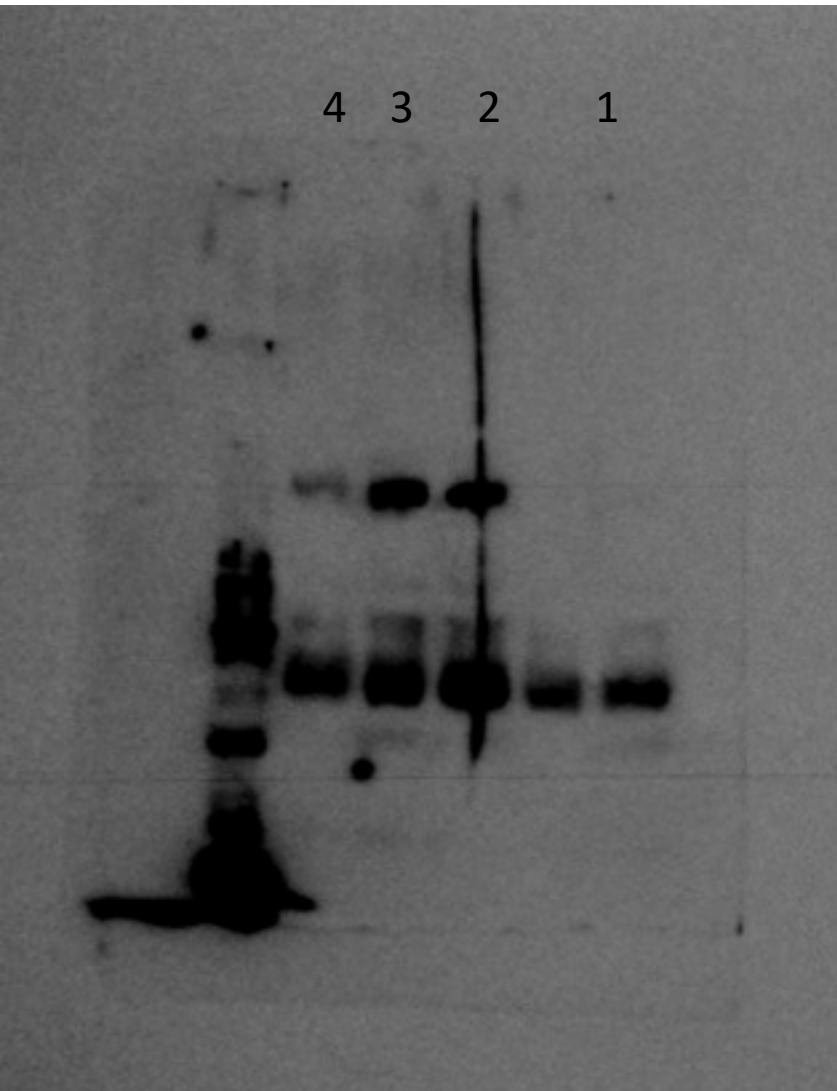

Not5 in Not4-AID

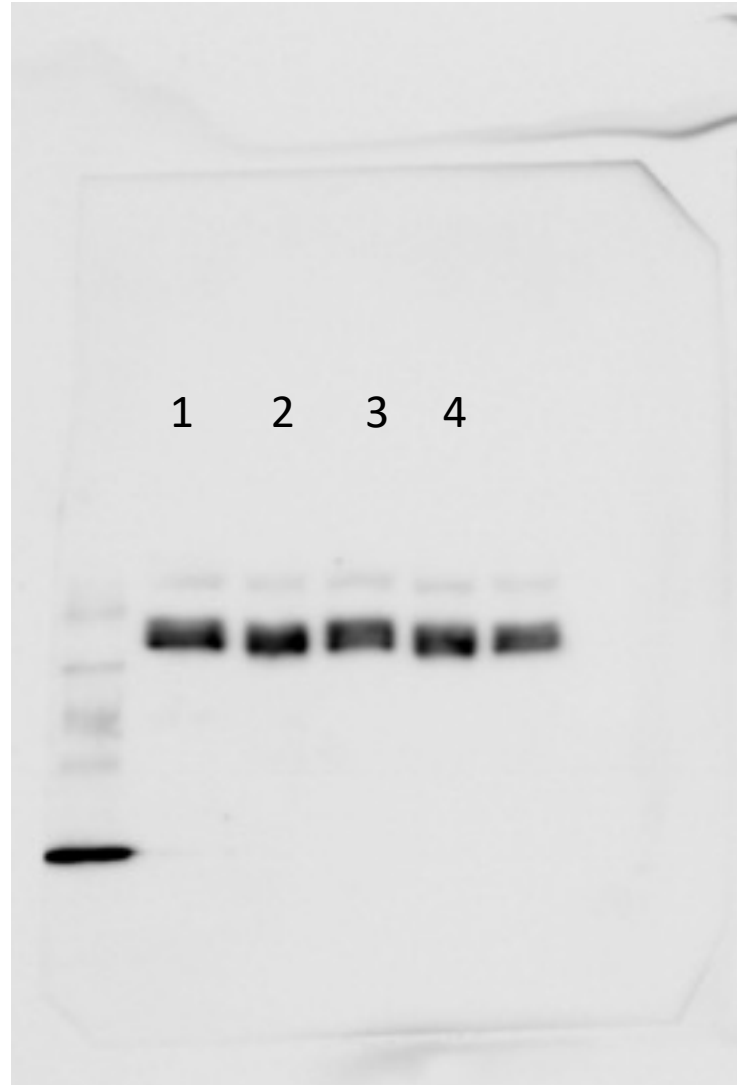

Supplement: Supplementary file 6 — Additional file 6. A separate pdf file entitled: Uncropped blots. Related to Figure S2A. [file 13059_2023_2871_MOESM6_ESM.pdf]
