# Supplementary material for: Not1 and Not4 inversely determine mRNA solubility that sets the dynamics of co-translational events
Source: Genome Biol. 2023 Feb 20;24:30. doi: 10.1186/s13059-023-02871-7 (PMC9940351; doi:10.1186/s13059-023-02871-7)
Supplement: Supplementary file 7 — Additional file 7. Review history. [file 13059_2023_2871_MOESM7_ESM.docx]

**1^st^ round**

**Reviewer 1**

This manuscript reports an analysis of mRNA populations defined by biochemical extraction procedures as "soluble" and "total" and of their degradation intermediates defined by 5'P-seq. These populations were analyzed in wild type yeast cells as well as in cells depleted of specific subunits of the CCR4-NOT complex.

Not surprisingly, and rather as expected, mRNAs extracted by different procedures show different characteristics, albeit limited (see below). Again, as one could have guessed, altering factors that broadly impact the transcriptome has an effect on these populations. Thus, the main issue is what new biological knowledge can we extract from these data? Unfortunately, the manuscript conclusions remain relatively vague on this issue. Not1 and Not4 are described at critical factors setting the dynamic of cotranslational events (see title) but again what this means is somewhat fuzzy.

Several issues are raised by the methods used. Two different methods are used to extract "total" RNAs. Yet it is unclear for which analysis one or the other was used. Does this contribute to the conclusions? Were the two methods shown to produce identical results? If so, these important data should be shown! What are the biological markers that validates the differential fractionations?
The strength of some data is also questionable: Commenting Figure 1B, the authors note that mRNA solubilities range from -2.744 to 1.791 (page 6) but are these extreme values significant? The distribution appears to be essentially a Gaussian (Figure 1B) and thus we may just look at random variations (?). Similarly, the comparison of total versus soluble mRNAs show that differences are very limited (Figure 1A). Where are the significant differences? Given the low signal, are biological triplicates sufficient to identify relevant differences? This seems unlikely.
Other analyses (e.g., correlations in Figure S2B) are described as highly significant, but visually poorly convincing (note R=0.12!!!). Else, the level of Not5 is described as unchanged upon Not4 depletion in the text but inspection of Figure 3A doesn't support this conclusion (compare the 15 min time points +/- auxin). The same issue occurs for Not4 level upon Not5 depletion. Finally, for some analyses arbitrary differences of 2 folds have been selected (Figure 2C) but whether those are significant or not remains unclear…

Overall, this manuscript presents a set of analyses that show differences in sets of mRNAs but it suffers from several technical limitations and more importantly fails to provide a clear biological explanation for the limited differences observed. If it is likely that translated mRNAs encoding membrane associated and secreted proteins are more difficult to extract than those encoding soluble proteins, many parameters may impact the observations reported in this manuscript. For example, are mRNAs encoding proteins that are imported or secreted only once completely translated equivalent to soluble mRNAs? Do mRNAs encoding membrane associated and secreted proteins use different codon overall than those for soluble proteins, in particular for rare codons? Do they have different amino-acid composition? Are they expressed at the same levels? Answering these questions may provide a biological bases to the differences observed.
In summary, I believe that the data presented in this manuscript are too preliminary and not sufficiently informative to warrant publication in Genome Biology.

Other points:
- The authors should clarify technical details: Which yeast strains were used for which experiments? Where they isogenic (WT strains aren't well described.) When was S. pombe mRNAs added to the different samples? (This may affect data normalization and result interpretations.)…

**Reviewer 2**

In this manuscript, the authors address how the conserved Ccr4-Not complex controls the global stability of soluble and insoluble mRNAs, the relationship this process has to Ccr4-Not mediated regulation of translation and the use of non-optimal codons, and how this process is integrated with Ccr4-Not transcriptional regulation. The authors provide data that the Not1 and Not4 subunits inversely affect the solubility of different mRNAs, and that the Not1-dependent regulation of this process may be dictated through Not1-dependent transcriptional control. They also provide evidence that Not4 can directly bind specific mRNAs, thus establishing for the first time that this component of the complex is a key RNA binding protein.
This manuscript provides a very thorough global analysis of Ccr4-Not mediated mRNA solubility, translation-coupled mRNA degradation, and the impact that global Not1 promoter binding and Not4 mRNA binding has with these processes. The data are of high quality and I believe the main points the authors make in this manuscript are well supported with the available data (there are some specific concerns outlined below). I would be supportive for this study to eventually be published in Genome Biology if the essential points highlighted below can be addressed.
The single largest concern with this manuscript relates to what feels like a lost opportunity for the authors to address an outstanding issue for the Ccr4-Not field. The authors provide experimental evidence that Not4 regulates specific classes of mRNA solubility, and that Not4 binds many of these mRNAs directly, which the authors rightly indicate is the first evidence provided in the literature that this subunit directly binds mRNA. However, it remains unclear whether the intrinsic Not4 ubiquitin ligase activity is required for the regulation of these functions, or if Not4's role in these processes is independent of its ubiquitin signaling activity. Because the authors used an auxin-inducible degron strain, it would have been very helpful to the field if they had done these experiments with the depleted strain reconstituted with either wild-type Not4 or a Not4 RING mutant that disrupts its ligase activity. These studies then would have allowed them to test directly whether Not4 mediated mRNA binding and its ubiquitin signaling control these post-transcriptional gene regulatory processes.

Essential points needing to be addressed
1) Related to the issue outlined above, I believe it would be helpful to the audience if the authors speculate whether there is a potential interrelationship between Not4 mRNA binding and regulation of mRNA solubility with Not4 mediated ubiquitin signaling. The authors have been pioneers in studying Ccr4-Not mediated ubiquitin signaling, having previously demonstrated that Ccr4-Not controls key aspects of proteasome regulation via Not4-mediated ubiquitin signaling and Ccr4-Not dependent co-translational proteasome assembly. It would be interesting to minimally speculate whether these processes are somehow integrated in vivo, ie. whether there is reason to believe that the soluble mRNA environment is "interpreted" by Not4 binding to dictate correct proteasome assembly and activity.
2) The authors use Not4 PAR-CLIP to demonstrate direct mRNA binding, but they do not do any experiments to directly determine which region of Not4 binds mRNA. Nor do they provide any context in the Discussion as to mechanistically suggest how Not4 may bind mRNA. Some elaboration on these issues would be helpful to further place the importance of these new data described in this manuscript within the diverse and essential functions controlled by Ccr4-Not.
3) On Page 9, lines 5-13, the authors discuss: "…that 1158 mRNAs exhibit opposing solubility changes in Not1 and Not4 depleted cells, with 637 less soluble on Not1 depletion but more soluble upon Not4 depletion, and 522 more soluble upon Not1 depletion and less soluble upon Not4 depletion." From the numbers listed in Fig. 2C, it is unclear where the authors are getting these values. This should be clarified as it is very confusing for the reader to understand given the large amount of data in this manuscript. Along these lines, any simplification of the text would help the reader interpret the large body of data presented in this paper.

**Reviewer 3**

The Ccr4-Not complex plays many roles during the translation process. In this study, Allen et al. demonstrated the inverse regulation of mRNA solubility by two factors in Ccr4-Not complex, named Not 1 and Not 4. By combining several different sequencing strategies, the authors addressed soluble mRNAs and insoluble mRNAs have different translation elongation dynamics and proposed a model that mRNA solubility sets the dynamics of co-translational events and is regulated by the Not proteins. These findings advanced our understanding of mRNA translation mechanism by modulation of solubility.

The manuscript was well written, and the figures are well presented. A minor concern needs to be addressed by the authors before publication. In figure 1A, the cutoffs used for setting mRNA groups with low or high solubility is confusing, which should be clarified for each analysis and be consistent to help compare the results from WT and Not1/4/5 depletion samples.

**Reviewer 4**

In this study the authors probe the role of Not1 and Not4 in influencing the partitioning of the soluble and insoluble (condensate) mRNAs and differences in translation properties and mRNA degradation rates between these two fractions. Using a range of tools, primarily different Seq methods and cross-linking, the authors show (1) that Not1 solubilizes a subset of mRNA's (i.e., pulls then out of condensates) while Not4 insolubulizes them and (2) the predominant mRNA degradation methods are different: insoluble mRNA tend to be degraded by co-translational pathways, while soluble mRNA tends to be degraded by non-translation dependent mechanisms. Overall, this is a very well written manuscript, with clear and reasonable methods, and data that supports their conclusions. I think the manuscript makes an important contribution by identifying differential degradatino behavior of mRNA in soluble versus insoluble fractions and how it is connected (or not) to co-translationnal processes. Therefore, I think the results are of broad interest to biologists.

I have no major nor minor concerns, and recommend publication.

**Authors’ response**

Reviewer #1:
This manuscript reports an analysis of mRNA populations defined by biochemical extraction procedures as "soluble" and "total" and of their degradation intermediates defined by 5'P-seq. These populations were analyzed in wild type yeast cells as well as in cells depleted of specific subunits of the CCR4-NOT complex.
Not surprisingly, and rather as expected, mRNAs extracted by different procedures show different characteristics, albeit limited (see below). Again, as one could have guessed, altering factors that broadly impact the transcriptome has an effect on these populations. Thus, the main issue is what new biological knowledge can we extract from these data? Unfortunately, the manuscript conclusions remain relatively vague on this issue. Not1 and Not4 are described at critical factors setting the dynamic of cotranslational events (see title) but again what this means is somewhat fuzzy.
Several issues are raised by the methods used.
Two different methods are used to extract "total" RNAs. Yet it is unclear for which analysis one or the other was used. Does this contribute to the conclusions? Were the two methods shown to produce identical results? If so, these important data should be shown! What are the biological markers that validates the differential fractionations?

Total RNA is extracted by acid phenol extraction of nucleic acids from cell pellets thus it contains all RNAs including insoluble RNAs. Soluble RNA is extracted after breaking cells with glass beads in a lysis buffer, centrifugation and then phenol extraction, thus it does not contain RNAs that were pelleted. This is detailed in the methods section (page 25). The content of each RNA pool is defined by RNA-Seq (total RNA of each pool). This is clarified in the text (page 6). The point of the manuscript is to compare these two RNA pools that have not been compared previously (and hence there are no biological markers for each pool). The interest of this comparison is that the soluble RNA pool is the one that methods such as ribosome profiling analyses, but it does not actually represent all cellular RNA pools and translation elongation profiles as our current study shows.

The strength of some data is also questionable: Commenting Figure 1B, the authors note that mRNA solubilities range from -2.744 to 1.791 (page 6) but are these extreme values significant? The distribution appears to be essentially a Gaussian (Figure 1B) and thus we may just look at random variations (?). Similarly, the comparison of total versus soluble mRNAs show that differences are very limited (Figure 1A). Where are the significant differences? Given the low signal, are biological triplicates sufficient to identify relevant differences? This seems unlikely.
To answer this point, we have replaced Figure 1A (new legend page 18,19), and as evident on this figure (see below), 21.5% of mRNAs are significantly changed...100*(665+539)/5609 (where 5609 is the number of mRNAs passing the minimum expression filter). Significant changes are defined by DeSeq2 as in Figure 2A (see methods). These values indicate that indeed, triplicate samples were sufficient to identify a large number of statistically significant differences.

Other analyses (e.g., correlations in Figure S2B) are described as highly significant, but visually poorly convincing (note R=0.12!!!).
We did not refer to the results mentioned by the reviewer as highly significant. What we precisely wrote about this figure is: “The depletion of Not5 exhibited marginal effects on the mRNA solubility and the subtle changes somewhat resembled those observed upon Not4 depletion (Figure S2B, left panel) but not those observed upon Not1 depletion (Figure S2B, right panel).” (page 8, bottom and page 9, top)
Perhaps the reviewer objects to the p-value, but this is simply the result of the most standard test of correlation:

"The test statistic is based on Pearson's product moment correlation coefficient R=cor(x, y) and follows a t distribution with length(x)-2 degrees of freedom if the samples follow independent normal distributions.”
One might question the assumption of an underlying Gaussian distribution in solubility, which the reviewers assesses themselves to be the case "The distribution appears to be essentially a Gaussian”. Equally, a Shapiro-Wilk normality test gives the following result:
W = 0.99717, p-value = 4.412e-08
So strong evidence for normality. Therefore, we can be confident that the significant p- value is mathematically sound. However, we do NOT draw the conclusion that not5d solubility levels are similar to not1d.
The reason the p-value is low because there are >5000 observations and thus >5000 degrees of freedom in the t-test.
Equally, we can’t find any instance in the entire manuscript of saying something is ‘highly/very significant'.

Else, the level of Not5 is described as unchanged upon Not4 depletion in the text but inspection of Figure 3A doesn't support this conclusion (compare the 15 min time points +/- auxin). The same issue occurs for Not4 level upon Not5 depletion.
We have done the auxin depletion experiments many times and there was no consistent change in any of the Not proteins except for the degron proteins at 15 min of auxin treatment. We have added this sentence in the main text (page 8) and in the figure legend (page 23). We agree with the reviewer that the blots produced were not the best to make the point as there was some uneven loading according to the Ponceau staining. For Not5 in the Not4 degron, we provide in the revised manuscript a different replicate of the experiment where the loading in all lanes was equal. For Not4 in the Not5 degron strain, since the signal for cells at 15 min with auxin and at 60 min without auxin was the same, we have left this experiment but added a comment in the figure legend about the loading (page 23). For the reviewers sake we include below an example of an experiment with the Not5 auxin degron mutant showing equal levels of Not4 upon Not5 depletion.

Question. Finally, for some analyses arbitrary differences of 2 folds have been selected (Figure 2C) but whether those are significant or not remains unclear...
To illustrate the selection of the oppositely regulated red and green solubility groups (Fig. 2C) based on significance, we have overlaid the genes (see attached figure) which are significantly changed based on a ratio-of-ratios test performed in DESeq2 (similar to a translational efficiency or RIP-Seq protocol) to compare solubilities. The purple lines represent the cutoffs we used for these groups. We see a handful of significant genes are below the cutoff, so to impose more stringent selection we filtered these, with very little loss. In this way, we now have our two focal groups of mRNAs which are oppositely regulated by Not1 and Not4, all significantly changed and distinct in fold change.
The orange and blue groups in Fig. 2C are then the opposite, selected as a type of control (by the same type of test) to have NO significant change between not1d and not4d in their solubility. This leaves a large control group which we wanted to split by a cutoff relative to WT, since we found that the (orange and blue) sub-groups behaved very distinctly from each other (expression, length, WT solubility, relative degradation, Not1RIP, Not4PAR-CLIP, Not1-ChEC). We also see that different functional groups are enriched in the blue and orange groups. We have added this explanation in the text (page 9).
Furthermore, for levels of most metrics (WT solubility, relative degradation, Not1RIP, Not4PAR-CLIP, Not1-ChEC), we see solubility changes due to not4d are the determining factor.
These inferences for the orange and blue groups could not be made using very small groups selected by significant changes to WT, so we used a cutoff leaving us with comparable numbers to the red and green groups. Despite the fact we principally focus on the red and green oppositely regulated transcript groups, we believe there would be a loss of information in removing their control groups or pooling all genes not oppositely regulated.

Overall, this manuscript presents a set of analyses that show differences in sets of mRNAs but it suffers from several technical limitations and more importantly fails to provide a clear biological explanation for the limited differences observed. If it is likely that translated mRNAs encoding membrane associated and secreted proteins are more difficult to extract than those encoding soluble proteins, many parameters may impact the observations reported in this manuscript. For example, are mRNAs encoding proteins that are imported or secreted only once completely translated equivalent to soluble mRNAs? Do mRNAs encoding membrane associated and secreted proteins use different codon overall than those for soluble proteins, in particular for rare codons? Do they have different amino-acid composition? Are they expressed at the same levels? Answering these questions may provide a biological bases to the differences observed.
In summary, I believe that the data presented in this manuscript are too preliminary and not sufficiently informative to warrant publication in Genome Biology.
The question raised by the reviewer is of course very interesting, namely the nature of the mRNAs with different solubilities and of those with inverse regulation by the Not proteins. We have looked at some parameters as provided in the manuscript. There are many other parameters that now can be investigated, and this is our current line of study, to understand according to which parameters the mRNA solubilities are regulated. However, this question extends beyond the scope of this manuscript that already has a very large body of data as mentioned by reviewer 2.

Other points:
- The authors should clarify technical details: Which yeast strains were used for which experiments? Where they isogenic (WT strains aren't well described.)
We have added the genotype of the strains used in materials and methods (page 25).
The isogenic wild type of the Not1, Not4 and Not5 degron strains were used with the degrons and the isogenic wild type of strain of the Not1-MNase strain was used with the Not1-MNase strain.

When was S. pombe mRNAs added to the different samples? (This may affect data normalization and result interpretations.)...
The spike in S. Pombe RNA was added to all RNA samples before they were split in half, one half used for RNA-Seq, one half of 5’P-Seq. This allowed the calculation of relative degradation for each RNA sample. This was explained in the results section and we have now added this description in the methods section of the revised manuscript (page 25, 26).

Reviewer #2:
In this manuscript, the authors address how the conserved Ccr4-Not complex controls the global stability of soluble and insoluble mRNAs, the relationship this process has to Ccr4-Not mediated regulation of translation and the use of non-optimal codons, and how this process is integrated with Ccr4-Not transcriptional regulation. The authors provide data that the Not1 and Not4 subunits inversely affect the solubility of different mRNAs, and that the Not1-dependent regulation of this process may be dictated through Not1-dependent transcriptional control. They also provide evidence that Not4 can directly bind specific mRNAs, thus establishing for the first time that this component of the complex is a key RNA binding protein.

This manuscript provides a very thorough global analysis of Ccr4-Not mediated mRNA solubility, translation-coupled mRNA degradation, and the impact that global Not1 promoter binding and Not4 mRNA binding has with these processes. The data are of high quality and I believe the main points the authors make in this manuscript are well supported with the available data (there are some specific concerns outlined below). I would be supportive for this study to eventually be published in Genome Biology if the essential points highlighted below can be addressed.
The single largest concern with this manuscript relates to what feels like a lost opportunity for the authors to address an outstanding issue for the Ccr4-Not field. The authors provide experimental evidence that Not4 regulates specific classes of mRNA solubility, and that Not4 binds many of these mRNAs directly, which the authors rightly indicate is the first evidence provided in the literature that this subunit directly binds mRNA. However, it remains unclear whether the intrinsic Not4 ubiquitin ligase activity is required for the regulation of these functions, or if Not4's role in these processes is independent of its ubiquitin signaling activity. Because the authors used an auxin- inducible degron strain, it would have been very helpful to the field if they had done these experiments with the depleted strain reconstituted with either wild-type Not4 or a Not4 RING mutant that disrupts its ligase activity. These studies then would have allowed them to test directly whether Not4 mediated mRNA binding and its ubiquitin signaling control these post-transcriptional gene regulatory processes.
We agree with the comments of the reviewer. Such studies would potentially be of high interest. However, to address them new strains need to be created and tested. This will launch an entirely new direction of study, which is beyond the scope of the current
manuscript.

Essential points needing to be addressed
Related to the issue outlined above, I believe it would be helpful to the audience if the authors speculate whether there is a potential interrelationship between Not4 mRNA binding and regulation of mRNA solubility with Not4 mediated ubiquitin signaling. The authors have been pioneers in studying Ccr4-Not mediated ubiquitin signaling, having previously demonstrated that Ccr4-Not controls key aspects of proteasome regulation via Not4-mediated ubiquitin signaling and Ccr4-Not dependent co-translational proteasome assembly. It would be interesting to minimally speculate whether these processes are somehow integrated in vivo, ie. whether there is reason to believe that the soluble mRNA environment is "interpreted" by Not4 binding to dictate correct proteasome assembly and activity.
As mentioned above, this is an exciting new idea proposed by the reviewer, which however extends beyond the scope of this manuscript.

2)The authors use Not4 PAR-CLIP to demonstrate direct mRNA binding, but they do not do any experiments to directly determine which region of Not4 binds mRNA. Nor do they provide any context in the Discussion as to mechanistically suggest how Not4 may bind mRNA. Some elaboration on these issues would be helpful to further place the importance of these new data described in this manuscript within the diverse and essential functions controlled by Ccr4-Not.
We have not done any experiments to address which domains of Not4 are key for the regulation of mRNA binding and solubility. We mention in the discussion that Not4 likely binds mRNAs already during transcription (page 17). Thereafter it may cycle on and off during translation as we also mention in the discussion (page 17), and it could be that the on state is promoted by Not4-interacting with ribosome-associated Not5.
We have added a sentence in the discussion with this speculative idea as suggested by the reviewer (page 17). To experimentally address these questions thoroughly, we would need to create a plethora of strains, a lengthy process that goes beyond the scope of our study.

3)On Page 9, lines 5-13, the authors discuss: "...that 1158 mRNAs exhibit opposing solubility changes in Not1 and Not4 depleted cells, with 637 less soluble on Not1 depletion but more soluble upon Not4 depletion, and 522 more soluble upon Not1 depletion and less soluble upon Not4 depletion." From the numbers listed in Fig. 2C, it is unclear where the authors are getting these values. This should be clarified as it is very confusing for the reader to understand given the large amount of data in this manuscript
We apologize, there was a mistake in calculation of the total of red and green mRNAs which is 1159 not 1158 (637 + 522). We have corrected this in the text.
We have also added clarification for how these values were obtained in the revision (page 9).

Along these lines, any simplification of the text would help the reader interpret the large body of data presented in this paper.
We are well aware that there is a large body of data in the manuscript that it takes time to integrate. We have really tried to be as simple as possible and have added some clarifications.

Reviewer #3:
The Ccr4-Not complex plays many roles during the translation process. In this study, Allen et al. demonstrated the inverse regulation of mRNA solubility by two factors in Ccr4-Not complex, named Not 1 and Not 4. By combining several different sequencing strategies, the authors addressed soluble mRNAs and insoluble mRNAs have different translation elongation dynamics and proposed a model that mRNA solubility sets the dynamics of co-translational events and is regulated by the Not proteins. These findings advanced our understanding of mRNA translation mechanism by modulation of solubility.
The manuscript was well written, and the figures are well presented. A minor concern needs to be addressed by the authors before publication. In figure 1A, the cutoffs used for setting mRNA groups with low or high solubility is confusing, which should be clarified for each analysis and be consistent to help compare the results from WT and Not1/4/5 depletion samples.
As mentioned in response to the comments to reviewer 1, we have changed figure 1A to be presented as Figure 2A, and calculated mRNA with significantly different solubility exactly as in Figure 2A, and this is now described both in the legend to Figure 1A (page 18,19) in addition to the methods.

Reviewer #4:
In this study the authors probe the role of Not1 and Not4 in influencing the partitioning of the soluble and insoluble (condensate) mRNAs and differences in translation properties and mRNA degradation rates between these two fractions. Using a range of tools, primarily different Seq methods and cross-linking, the authors show (1) that Not1 solubilizes a subset of mRNA's (i.e., pulls then out of condensates) while Not4 insolubulizes them and (2) the predominant mRNA degradation methods are different: insoluble mRNA tend to be degraded by co-translational pathways, while soluble mRNA tends to be degraded by non-translation dependent mechanisms. Overall, this is a very well written manuscript, with clear and reasonable methods, and data that supports their conclusions. I think the manuscript makes an important contribution by identifying differential degradatino behavior of mRNA in soluble versus insoluble fractions and how it is connected (or not) to co-translationnal processes. Therefore, I think the results are of broad interest to biologists.
I have no major nor minor concerns, and recommend publication.
We thank this reviewer for their supportive comments.

**2^nd^ round**

**Reviewer 1**

In this revised version of their manuscript, authors present some answers to issues raised by reviewers.
Concerning the method(s) used to extract total mRNAs and the concern I had with two different protocols, the authors indicate that a single acid phenol mRNA extraction method was used. Yet, the Material and Methods section indicates (page 25, lines 35-37): “Total RNA was prepared either by the hot acid phenol method (71) or cells were prepared and lysed as for polysome profiling (42) and total RNA was prepared from the lysate.”. Thus, either the Material and Methods is erroneous or my issue with how total mRNAs extracted by two protocols compare remains unanswered. This point remains problematic.
Yet, the main argument presented by the authors to answer concerns about the biological value of their data is statistical analyses. For example, authors addressed one of this point by changing Figure 1A, illustrating mRNAs they argue are significantly changed. Statistical validity is based on DESeq2. However, the latter package was designed to analyze differential expression between independent samples and the authors fail to justify why these statistics would apply to compare paired samples (soluble versus total) that aren’t independent (soluble is a fraction of total)? The validity of DESeq2 for this type of analysis is indeed further questioned by Fig. S2B in which poor correlations (e.g., R=0.12) have extraordinary p-value (9e-18!). But again, assumption for the statistical handling of these data are certainly incorrect: the authors indicate in their answer that the treatment used implies “independent normal distributions” but given that the two distributions compared are both normalized with WT, data they aren’t independent. Moreover, the degree of freedom used for the t-test is questionable. Altogether, the statistical treatment of the data is at best puzzling. Nevertheless, I agree with the authors that they aren’t “saying something like ‘highly/very significant’” (see their answer to reviewers’ comments) but I wonder whether the absence of such statement just confirms my comments and is thus a counter-argument for publication in Genome Biology!

Beside this main concern, several other issues remain problematic:
- Given the protocols used to extract total mRNAs and “soluble” mRNAs, it remains possible that authors are only observing a modest impact of cycloheximide, magnesium and/or EDTA rather on mRNA extraction/degradation rather than mRNP solubility. Their interpretation referring only to this parameter is not sufficiently justified.
- How other mRNA decay pathways (e.g., 3’-5’), that may be specifically activated upon depletion of one or the other NOT protein, may interfere with the reported analyses?
- In Material and Methods, authors mention several plasmids without description or reference. This isn’t useful. Tables with all strains and plasmids would be valuable.
- Page 26: typo “spited”
- Page 27 mentions NOT5- MNAse and REB1-MNAse fusions that aren’t describe in the yeast strains nor presented in results.
- Page 28: typo “changed tRNAs”

Altogether, I stand by my original conclusion, finding limited biological information in the revised manuscript. I don’t recommend its publication in Genome Biology.

**Reviewer 4**

I think the manuscript is sound and interesting. I support publication of this manuscript.

Due to the disagreement between Reviewer 1 and the authors, and the generally positive comments of the other reviewers, the editors invited an additional reviewer to comment specifically on the statistical concerns of Reviewer 1.

**Reviewer 5**

Out of the concerns raised by the reviewer, the one we find potentially serious is "given that the two distributions compared are both normalized with WT, data they aren’t independent”. However, we believe this is a result of poor clarify from the authors when using the term WT. Their use of the term makes an impression that they are normalising their two lines to a third, genuinely “wild-type” strain - and this is what reviewer4 understands. However, reading the Fig 2/3 captions, it is more likely that they are using the same strains prior to knockdown (i.e, prior to auxin). It’s essential that they clarify this and arguably they should rename WT to “before/after auxin”, or something like this. These lines are NOT “wild-type”. Conversely, if they were actually normalising to a third WT line as reviewer4 understands, this wouldn’t be acceptable, as it would lead to false positive +ve correlations.

We are not convinced by the remaining criticisms of reviewer4. Their comment demonstrates a poor understanding of p values, we don’t agree with their criticisms of DESeq2, and also the fact the distributions look Gaussian doesn’t mean it’s just noise and not significant or biologically relevant.

Regarding questions on extraction biases due to different methods. It would be good if the authors could fully clarify. Such biases could impact results for a single condition, but one expects that they should “cancel out” when comparing between mutants (so comparisons (eg 2c) could be valid). They could perhaps add some caution to general claims like in the abstract “insoluble mRNAs are enriched for ribosomes dwelling at nonoptimal codons compared to soluble mRNAs” - as they could be partly of technical nature

**Authors’ response**

Given the protocols used to extract total mRNAs and "soluble" mRNAs, it remains possible that authors are only observing a modest impact of cycloheximide, magnesium and/or EDTA rather on mRNA extraction/degradation rather than mRNP solubility. Their interpretation referring only to this parameter is not sufficiently justified.
Because of the confusion that remains concerning the total RNA (soluble and insoluble ) and the total soluble RNA (only soluble) we have chosen to rename the total RNA extracted by acid phenol: the “total RNA” and the RNA extracted by bead beating in a lysis buffer the “soluble RNA pool”. This should avoid the confusion of using the word “total” for 2 different RNA pools.
We have added a comment in the manuscript to caution that obviously it can be expected that the “soluble RNA pool” can be different depending upon the salt and detergent conditions used in the lysis buffer. We used the conditions typically used for polysome and ribosome profiling as this was really the point discussed in the manuscript, namely that what is typically measured by these approaches, is only a partial view of translating mRNAs in the cell.
How other mRNA decay pathways (e.g., 3'-5'), that may be specifically activated upon depletion of one or the other NOT protein, may interfere with the reported analyses?
We do not measure the contribution of 3’ to 5’ decay pathways in this work since we follow 5’P decay intermediates, so we can only speculate. However, we have already made such a speculation concerning the deletion of Not5 in a previous study in which we mentioned that the assembly of the exosome (3’ to 5’ decay) is impaired in cells lacking Not5 (Allen et al., 2021, Cell Reports) and even though it is induced (Azzouz et al., 2009, RNA), it is not clear that its contribution would be increased.
Without many additional very specific experiments we really cannot say more and hence we do not see that it is relevant to add a comment in this regard.

In Material and Methods, authors mention several plasmids without description or reference. This isn't useful. Tables with all strains and plasmids would be valuable.
We have added a table (Table S5) listing strains and plasmids.

Page 26: typo "spited"
We have corrected the typo.

Page 27 mentions NOT5- MNAse and REB1-MNAse fusions that aren't describe in the yeast strains nor presented in results.
The references have been provided.

Page 28: typo "changed tRNAs"
We have corrected the typo.
The Major comment of reviewer 1 has been addressed by Reviewer 5:

Reviewer #5 (comments on Reviewer #1):
Out of the concerns raised by the reviewer, the one we find potentially serious is "given that the two distributions compared are both normalized with WT, data they aren’t independent”. However, we believe this is a result of poor clarify from the authors when using the term WT. Their use of the term makes an impression that they are normalising their two lines to a third, genuinely “wild-type” strain - and this is what reviewer4 understands. However, reading the Fig 2/3 captions, it is more likely that they are using the same strains prior to knockdown (i.e, prior to auxin). It’s essential that they clarify this and arguably they should rename WT to “before/after auxin”, or something like this. These lines are NOT “wild-type”. Conversely, if they were actually normalising to a third WT line as reviewer4 understands, this wouldn’t be acceptable, as it would lead to false positive +ve correlations.
We are not convinced by the remaining criticisms of reviewer4. Their comment demonstrates a poor understanding of p values, we don’t agree with their criticisms of DESeq2, and also the fact the distributions look Gaussian doesn’t mean it’s just noise and not significant or biologically relevant.
Concerning this remaining concern "given that the two distributions compared are both normalized with WT, data they aren’t independent”, and possible “false positive +ve correlations” we reply the following:
Addressing the comments of reviewer #1 along with the comments of the new reviewer #5, we realise that an imprecise use of “normalized” when referring to a comparison between the degrons and the wild type might be in the core of misunderstanding and criticism. We certainly apologize for the confusion.
We show “difference” or “change” in solubility (Figure 2) or “relative degradation” (Figure 3) in the degron strains after depletion of the degron protein relative to the isogenic wildtype strain with the TIR, but without the degron allele, both treated with auxin. We do not normalize the values of the degron strains and only compare to the isogenic wild type strain.
Otherwise, the reviewer is correct, that if normalization would be undertaken, then it is correct to normalize on the closest condition, i.e. here with degron without auxin treatment, to not amplify the effect of changes driven by degron itself.
We have modified the indications on the figure axes accordingly and clarified in the text. We compare the effect of depleting one or a few different proteins to the cognate wild type strain;
thereby, all are expressing TIR and the comparison is performed after auxin treatment. In our opinion such a comparison captures the biological meaning of this experiment, namely to exclude any effect the degron itself may have. Unlike tagging of proteins where the effect persists in the cell, degron- tagging enables controllable degradation and hence, lack of (protein) effect. Thus, a comparison to a silent degron may indeed report on the effect on the degron itself, which in turn is gone when treating with auxin. Thus, we believe that using the wild type, TIR-containing strain, which was used to make the degron strains, for comparison to the different degron strains after auxin treatment, removes any problem related to the degron tagging itself.
This approach is widely accepted in the field, has been used to characterize the function of individual proteins (eg eiF5A by the Green lab: doi: 10.1016/j.molcel.2017.03.003), and our reasoning for comparison of several degrons strains was supported by already published examples with degron strains (doi:https://doi.org/10.1016/j.molcel.2018.10.018). Please note that the authors compare multiple degrons – as we do – only to the same parental TIR-containing strain after auxin treatment.

As supported by the comments of Reviewer #5 we provide below some explanations supporting the appropriate use of DESeq2. We believe it is an entirely appropriate package and has been used in similar ways routinely in literature. We compare solubility between strains using a ratio-of-ratios test performed in DESeq2 (the ratios compared being soluble/total within each strain and hence, not requiring a common normalization). This is identical to a test of polysome/total RNA-Seq for two conditions (the polysome being a subset of the total) – a test largely established in the literature. We include below an example from a published paper in support of this.

Ref: Vaishnavi Shankar, Robert Rauscher, Julia Reuther, Walid H Gharib, Miriam Koch, Norbert Polacek, rRNA expansion segment 27Lb modulates the factor recruitment capacity of the yeast ribosome and shapes the proteome, Nucleic Acids Research, Volume 48, Issue 6, 06 April 2020, Pages 3244– 3256, <https://doi.org/10.1093/nar/gkaa003>

Similar to the above example, in our most important DESeq2 comparison, we compare solubilities in
not1d and not4d degron strains and require that the ratio-of-ratios (the ratios compared being soluble- to-insoluble within each strain) test shows a significant difference between strains. We also require solubility in both degron strains is different from WT. This decision to compare both to WT is to make sure that the selected transcripts are oppositely regulated by Not1 and Not4; for instance, if a transcript’s solubility does not change from WT in not1d it still may have a significantly different solubility in not4d but its solubility may not be regulated by Not1. We want transcripts where solubility will increase when Not1 is lost and decrease when Not4 is lost (or vice-verse) to examine
the possibility of antagonistic regulation.
The orange and blue groups in Fig. 2C are then the opposite, selected as a type of control (by the same type of test) to have NO significant change between not1d and not4d in their solubility. This leaves a large control group which we wanted to split by a cutoff relative to WT, since we found that the (orange and blue) sub-groups behaved very distinctly from each other (expression, length, WT solubility, relative degradation, Not1RIP, Not4PAR-CLIP, Not1-ChEC) Again supported by the comments of Reviewer #5, we maintain that a low R-value for correlation can still result in a highly significant p-value when comparing values for thousands of transcripts. The low p-value results from the high degrees of freedom n-2 where n>5000. We include a below direct two- tailed test of the relevant t-statistic to illustrate unambiguously that this is accurate:
> r=0.12
> n=5083
> pt(r*sqrt((n-2)/(1-r^2)), df=n-2,lower.tail=FALSE)*2
[1] 9.135134e-18
The degrees of freedom for correlations are the total number of score pairs (n) minus two. There is no other possibility. Nevertheless, we believe that p-values in these cases are not very useful for interpretation and base our conclusions on the R-value. Indeed, in the very first submission, we wrote of the right panel where R=0.12:
‘The depletion of Not5 exhibited marginal effects on the mRNA solubility and the subtle changes somewhat resembled those observed upon Not4 depletion (Figure S2B, left panel) but not those observed upon Not1 depletion (Figure S2B, right panel).’

In support of the comment that “the fact the distributions look Gaussian doesn’t mean it’s just noise and not significant or biologically relevant”, we contend that it not unusual that RNA-Seq fold change distributions between samples are Gaussian. Equally, we see the range of log2 fold changes is similar in the figure included above for the polysome profiling paper.
Regarding the issue of potential “false positive +ve correlations”, we would like to clarify/underline that this does not apply to the negative correlation observed in Fig. 2C. Systematic random variation in the WT cannot lead to false negative correlation of fold changes over WT. This means that in Fig. 2C, where changes in solubility due to Not1 and Not4 depletions are negatively correlated, it is impossible that the extent of this negative correlation could be exaggerated by random variation in a common baseline:

This is because any random loss in WT solubility will result in an increase in ‘change in solubility’ for
both not1d and not4d, since we are subtracting a smaller value for both. (Equally, any random gain in WT results in loss in ‘change in solubility’ for both strains). Of course, a random change in WT such as this cannot make the solubility changes calculated for each strain more opposite, only more similar, if at all. This means, random changes in WT can only increase an R value, not reduce it. Therefore, no negative correlation could be exaggerated in this way.
To illustrate this concept, we produced 1000 simulations in which we added noise to the original WT solubility value for each transcript, sampled from a uniform distribution on [-0.15,0.15] and, for each simulation, we calculated new R values correlating changes in solubility for Not1 and Not4, each relative to the new WT (i.e. the original WT with noise added).
A histogram of the 1000 resulting R values is included below. We can clearly see that, in every case, the new R values are all greater than the original (albeit only slightly). This shows that, the anti- correlation between changes in solubility due to Not1 and Not4 depletion is as least as strong as we present. Therefore, we believe this figure should be maintained since the opposite changes in solubility are not exaggerated by the common baseline (the contrary, if anything) and it is much more informative to be able to illustrate how the Not1 and Not4 degrons lead to inverse solubility effects relative to WT.

More broadly, since we have triplicates in soluble and total and to obtain solubility we are pooling
these and taking a ratio with DESeq2 (with expression filters), we believe random, non-biological variation in the WT should be low.
Regarding plots of positive correlations, there are examples in the literature of (positively) correlating fold changes against a common baseline. We include one below to illustrate:

Ref: Veetil RT, Malhotra N, Dubey A, Seshasayee ASN. Laboratory Evolution Experiments Help Identify a Predominant Region of Constitutive Stable DNA Replication Initiation. mSphere. 2020 Feb 26;5(1):e00939-19.
doi: 10.1128/mSphere.00939-19. PMID: 32102945; PMCID: PMC7045392.
<https://doi.org/10.1128/mSphere.00939-19>

Another way to exemplify this point is that each particular mRNA has some intrinsic solubility (due to their translation, structure, regulation...). And this intrinsic solubility will generate always a positive correlation across samples. However, the intrinsic solubility is modified by their interaction with Not1 and Not4, that is the focus of this paper. Thus, to focus on the biology of how Not1 and Not4 control mRNA solubility, one needs to refer the changes in solubility to the control strain. This same point can be exemplified when talking about mRNA association to polysomes, something that is commonly analysed in the literature.
There is one other correlation plot in the main text (Fig. 3B), which shows a positive correlation. To examine alternatives, we plotted the positive correlation of changes in degradation rate in the degrons relative to WT (left panes below) and compared it side-by-side to direct correlation of degradation rate in the degrons:

This shows that there is a reasonably large increase in correlation when not subtracting the WT and the transcripts distinguished by solubility separate in the same way. This is because, generally, transcripts with a high degradation rate in WT have a higher rate in the both strains with activated degrons, and those with a low rate are lower in the degrons. We are concerned that a direct comparison exaggerates the similarity in how the two depletions affect degradation rate.
Aside from the main text, there are two panels in Fig. S2B showing positive correlations of differences in solubility comparing Not1 and Not4 depletions to Not5 depletion. In the manuscript, we comment on them in the following way:
‘The depletion of Not5 exhibited marginal effects on the mRNA solubility and the subtle changes somewhat resembled those observed upon Not4 depletion (Figure S2B, left panel) but not those observed upon Not1 depletion (Figure S2B, right panel).’
Again, we compared correlations between strains when subtracting WT solubility to give change in solubility relative to WT (left panels) vs direct correlation of solubility between strains without subtracting WT (right panels).
The big change we see is comparing Not1 and Not5 degrons, where the R value increases from 0.12 to 0.56, since the solubility in the Not1 depletion and Not5 depletion strains correlate quite well with WT, but while Fig. 2A illustrates that not1d shows strong changes (soluble transcripts get significantly more soluble, insoluble transcripts get significantly less soluble), we see that Not5 depletion has little significant effect on solubility levels. We aim to show there is little association between the pattern in solubility changes arising from Not1 and Not5 depletions and were concerned that a direct correlation is too convoluted with WT patterns to show this.

Another way to visualize this is to plot the Not1 and Not5 depletions against the wild type separately:

Overlaying genes changing in solubility in not1d (top row) or not4d (bottom row) again, show clearly that there is no association between changes in solubility levels in Not1 and Not5 depletions and some association between changes in Not4 and Not5 depletions. We believe the loss here is that we do not immediately see, for instance, that a higher change in Not4 depletion, usually implicates a higher change in Not5 depletion.

Reviewer 5 last comment:
Regarding questions on extraction biases due to different methods. It would be good if the authors could fully clarify. Such biases could impact results for a single condition, but one expects that they should “cancel out” when comparing between mutants (so comparisons (eg 2c) could be valid). They could perhaps add some caution to general claims like in the abstract “insoluble mRNAs are enriched for ribosomes dwelling at nonoptimal codons compared to soluble mRNAs” - as they could be partly of technical nature
We agree with this comment and as mentioned in response to reviewer 1, we have added a word of caution concerning the “soluble RNA pool” (pages 7,8).
